# Identification of Ser/Thr kinase and Forkhead Associated Domains in *Mycobacterium ulcerans:* Characterization of Novel Association between Protein Kinase Q and MupFHA

**DOI:** 10.1371/journal.pntd.0003315

**Published:** 2014-11-20

**Authors:** Gunjan Arora, Andaleeb Sajid, Anshika Singhal, Jayadev Joshi, Richa Virmani, Meetu Gupta, Nupur Verma, Abhijit Maji, Richa Misra, Grégory Baronian, Amit K. Pandey, Virginie Molle, Yogendra Singh

**Affiliations:** 1 CSIR- Institute of Genomics and Integrative Biology, Delhi, India; 2 Translational Health Science and Technology Institute, Gurgaon, India; 3 Laboratoire de Dynamique des Interactions Membranaires Normales et Pathologiques, Universités de Montpellier II et I, CNRS, UMR 5235, Place Eugène Bataillon, Montpellier, France; University of Tennessee, United States of America

## Abstract

**Background:**

*Mycobacterium ulcerans*, the causative agent of Buruli ulcer in humans, is unique among the members of *Mycobacterium* genus due to the presence of the virulence determinant megaplasmid pMUM001. This plasmid encodes multiple virulence-associated genes, including *mup011*, which is an uncharacterized Ser/Thr protein kinase (STPK) PknQ.

**Methodology/Principal Findings:**

In this study, we have characterized PknQ and explored its interaction with MupFHA (Mup018c), a FHA domain containing protein also encoded by pMUM001. MupFHA was found to interact with PknQ and suppress its autophosphorylation. Subsequent protein-protein docking and molecular dynamic simulation analyses showed that this interaction involves the FHA domain of MupFHA and PknQ activation loop residues Ser^170^ and Thr^174^. FHA domains are known to recognize phosphothreonine residues, and therefore, MupFHA may be acting as one of the few unusual FHA-domain having overlapping specificity. Additionally, we elucidated the PknQ-dependent regulation of MupDivIVA (Mup012c), which is a DivIVA domain containing protein encoded by pMUM001. MupDivIVA interacts with MupFHA and this interaction may also involve phospho-threonine/serine residues of MupDivIVA.

**Conclusions/Significance:**

Together, these results describe novel signaling mechanisms in *M. ulcerans* and show a three-way regulation of PknQ, MupFHA, and MupDivIVA. FHA domains have been considered to be only pThr specific and our results indicate a novel mechanism of pSer as well as pThr interaction exhibited by MupFHA. These results signify the need of further re-evaluating the FHA domain –pThr/pSer interaction model. MupFHA may serve as the ideal candidate for structural studies on this unique class of modular enzymes.

## Author Summary

*Mycobacterium ulcerans* is a slow growing pathogen, which is prevalent in many tropical and sub-tropical countries. *M. ulcerans* possesses unique signaling pathways with only 13 STPK containing genes. This is strikingly different from its closest homolog *Mycobacterium marinum* and surprisingly closer to the human pathogen, *Mycobacterium tuberculosis*. PknQ, MupFHA and MupDivIVA are regulatory proteins encoded by the virulence determining plasmid pMUM001 of *M. ulcerans*. In addition to characterizing the STPK, we focused on deciphering the basis of interaction between the three partner proteins leading to the identification of critical residues. Present study describes the newly identified phosphoserine-based interactions, which is unique amongst the FHA-domain containing proteins. We confirmed our results using structural analysis via specific mutants and their interaction profiles. Importantly, these data highlight the significance of FHA domains and their role in understanding cellular signaling. This work will encourage further studies to elucidate role of *M. ulcerans* signaling systems. It will also raise questions like how less studied tropical bacterial pathogens acquire eukaryotic-like Ser/Thr protein kinase and exhibit unusual mechanisms to interact with its partner domains.

## Introduction

Buruli ulcer is a disease of skin and soft tissues caused by the bacteria *Mycobacterium ulcerans* [1]. It is the third most important mycobacterial disease after tuberculosis and leprosy [2], and the prevalence continues to increase in tropical and sub-tropical countries [3]. *M. ulcerans* evolved from an *Mycobacterium marinum* ancestor through reductive evolution and acquired a large virulence determinant plasmid (pMUM001) [4]. This plasmid encodes genes for mycolactone synthesis that are required to circumvent the host immune response, as a strain lacking this plasmid is avirulent. Therefore, the pMUM001 plasmid is considered to be a key determinant of *M. ulcerans* pathogenesis [5], [6].

Pathogenic species of mycobacteria require stringent control on cell division for survival in their host, and thus are likely to acquire specialized mechanisms through evolution to achieve this control. Signaling proteins that sense environmental changes and mediate cell response are important for regulating cell division. For instance, bacterial Serine/Threonine Protein Kinases (STPKs) are known to regulate cell division by sensing and responding to specific signals in the host environment [7]. Moreover, according to phospho-proteome analysis, numerous Ser/Thr phosphorylated proteins have been identified in *Mycobacterium tuberculosis*, suggesting that STPKs may regulate multiple cellular processes [8]–[12]. Indeed, eleven STPKs have been identified in *M. tuberculosis* and the majority of them have been shown to be involved in pathogenesis and drug resistance [13]–[15].

ForkHead-Associated (FHA) domain containing proteins are the key interacting partners of STPKs that mediate the signals inside the cells emanating from the cognate kinases [16]–[18]. FHA domains are highly conserved modules, known to bind phosphorylated residues within the proteins involved in diverse processes in bacteria, such as protein secretion, antibiotic resistance, transcription, peptidoglycan synthesis, metabolism and virulence [17]–[20]. Most of the FHA domain containing proteins in *M. tuberculosis* are phosphorylated by STPKs [18], and these proteins have been shown to recruit several other proteins in addition to being STPK substrates [17], [18], [21].

Based on the importance of STPKs in *M. tuberculosis* physiology and virulence, we explored the molecular transducers and their associated FHA domains in the *M. ulcerans* genome. We identified 13 STPK-encoding genes *in silico* and focused on a novel STPK- PknQ, encoded by the virulence-associated plasmid pMUM001. We identified MupFHA (Mup018c) and MupDivIVA (Mup012c) as the substrates of PknQ and characterized the interaction of PknQ and MupDivIVA with MupFHA. MupFHA contains one FHA domain that interacts with PknQ, as well as with phosphorylated MupDivIVA. Importantly, we found that the interactions of these three *M. ulcerans* proteins encoded by the virulence-associated plasmid are dependent on phosphorylation of serine and threonine residues.

## Methods

### Bioinformatic analyses

BLAST search was performed using the NCBI-BLASTp (http://blast.ncbi.nlm.nih.gov/Blast.cgi) with proteomes of *M. ulcerans* and *M. marinum* as target and desired protein sequence as query. The sequences of *M. ulcerans* STPKs were extracted using NCBI. These sequences were used for multiple sequence alignments, performed using ClustalW (http://www.ebi.ac.uk/Tools/msa/clustalw2). Phylogenetic analysis was performed using the sequences of STPKs in PHYLIP and phylogenetic tree was constructed [22]. The presence of conserved domains was detected by NCBI- conserved domain database (http://www.ncbi.nlm.nih.gov/Structure/cdd/cdd.shtml).

### Homology modeling

To generate the structure of PknQ, intracellular kinase domain of *M. tuberculosis* PknB (PDB ID: 1MRU) that shows 42% identity with the PknQ sequence, was chosen as template. We focused on the predicted catalytic domain of PknQ (1 to 280 aa). The three-dimensional models for PknQ were generated using MODELLER version 9v1. General features were evaluated on the basis of the MODELLER's energy and DOPE scores while detailed reliability indices were obtained by the PROCHECK program. The PROSA Z-score was calculated using PROSA-II. Best models were chosen and further refinements were carried out. Disordered activation loop region was identified and loop refinement procedure was applied using the automatic loop refinement method provided in MODELLER 9v1 auto-model class protocol. In order to model the loop reliably, 500 loop models were sampled followed by model validation using DOPE scores and Verify 3D. PTM-Viena server was used to modify the protein and phosphate group was added at 2 different positions- Ser^170^ and Thr^174^. *Ab initio* loop modeling was further used to minimize the post-translation modification effects. For this purpose, loop modeling protocol of standalone version of Rosetta 3.4 modeling suite was used and 50 outputs were taken [23]. On the basis of energy score and visual inspection of the loop we identified top 3 best loop models and used for further studies.

Similarly, structural modeling of the FHA domain was performed with MODELLER 9v1. MupFHA protein sequence spanning the residues 10 to 100 was modeled (containing the FHA domain and the interaction motif). In order to use the maximum benefits of sequence identities and to model the maximum part of sequence contacting FHA domain, multi-template approach provided by MODELLER 9v1 was utilized and the two templates of Rv0020c FHA domain (PDB ID: 2LC1, 3PO8) were used to predict the three dimensional homology model of MupFHA. The 3D models for native FHA domain and mutants were generated, on the basis of previously generated structures of Rv0020c and pThr peptide [24], [25]. The templates were pair-wise structurally aligned. Further models were built and validated as described for PknQ homology modeling.

### Protein docking

Protein-protein dockings were performed using HEX 6.3 molecular docking program, correlation type were chosen as shape and 2000 solutions were chosen for final evaluation. Other parameters were chosen as default. Structures of the wild type FHA domain and mutants were used for docking with PknQ containing pSer^170^ and pThr^174^. First 10 solutions were analyzed for each docking and best docked poses were chosen. A short minimization was performed with Gromacs to minimize the complexes. Intermolecular interactions and docking scores were analyzed.

### Molecular dynamics (MD) simulation

Three complexes were prepared using molecular dynamics simulation- (1) a double phosphorylated PknQ-FHA complex, (2) mutant complex having single phosphorylation on Ser^170^ and mutation at pThr^174^ to alanine (PknQ-pSer170/Thr174Ala), (3) phosphorylation at Thr^174^ and mutation at pSer^170^ to alanine (PknQ-Ser170Ala/pThr174). All the simulation studies were performed on GROMACS 4.5.5 using ffG43a1p force field provided by Gromacs official website (http://www.gromacs.org/Downloads/User_contributions/Force_fields) and uploaded by Graham Smith, which included extended parameters for modified residues [26].

The protein complex was dissolved at the center of a cubic box, solvated with single point charge water molecules. Solute-box distances of 1.0 nm were specified. To simulate the protein-protein complex system and in order to solve the issue of surface effects, periodic boundary conditions were applied. To neutralize the net charge on the protein, Na^+^ ions were added. A short energy minimization step was performed on the solvated system with steepest descent algorithm for 50000 steps.

Two phases of equilibration were conducted for 100 ps each- under constant Number of particles, Volume and Temperature (NVT) followed by constant Number of particles, Pressure and Temperature (NPT). Positional restraints were applied on the protein to allow solvent molecules to relax around the structure. In the second stage, positional restraints were lifted and the system was coupled to a heat bath at 300 K using the Berendsen thermostat and allowed to equilibrate for 100 ps [27]. In an NPT ensemble, the Parrinello-Rahman barostat was used for controlling the pressure [28]. Time constants for controlling the temperature and pressure were set to 0.1 ps and 2 ps, respectively.

The production run was performed with suitable parameters for 10 ns at a temperature and pressure of 300 K and 1 atm, respectively. Coordinate sampling was performed at every 2 fs time interval. Bond-lengths were constrained using the Linear Constraint Solver (LINCS) algorithm [29]. Various utilities of GROMACS-4.5.5 package were used for detailed analysis of MD trajectories. All of the Gromacs MD simulations were run in the HPC-Supercomputer facility (CSIR-IGIB, India) on 32 Cores at 11.4 ns/day maximum performance speed.

### Bacterial strains and growth conditions

*Escherichia coli* strain DH5α (Novagen) was used for cloning and BL21 (DE3) (Stratagene) for the expression of recombinant proteins. *E. coli* cells were grown and maintained with constant shaking (200 rpm) at 37°C in LB broth supplemented with appropriate antibiotic (100 µg/ml ampicillin and/or 12.5 µg/ml chloramphenicol), when needed.

### Gene cloning and generation of site-directed mutants

For cloning of *pknQ_fl_* (*mup011*, 1–660 amino acids), its catalytic kinase domain *pknQ_kd_* (1–344 aa), *mup018c* (1–362 aa) and *mup012c* (1–87 aa), the respective genes were amplified by PCR from *M. ulcerans* Agy99 plasmid pMUM001 using specific primers (Table S1). The resulting PCR products were cloned into the selected vectors (pProEx-HTc, pGEX-5X-3, pMAL-c2x and/or pACYCDuet-1). *Rv0020c* was PCR amplified from *M. tuberculosis* H37Rv genomic DNA and cloned in pProEx-HTc vector. The plasmid derivatives were confirmed with restriction digestion and DNA sequencing (Invitrogen) (Table S1). *M. tuberculosis* PstP (Rv0018c) and *Bacillus anthracis* PrkD/PrkD^S162A^ were cloned as described earlier [30], [31].

To generate the specific site mutants (Table S1) of PknQ, MupFHA and MupDivIVA, site directed mutagenesis was carried out using QuikChange XL Site-Directed Mutagenesis Kit (Stratagene) according to the manufacturer's protocol. The mutants were confirmed by DNA sequencing.

### Expression and purification of recombinant proteins

The plasmids were transformed and proteins were over-expressed in *E. coli* BL21 (DE3). The recombinant GST-tagged fusion proteins were affinity purified with glutathione sepharose column. The His_6_-tagged proteins were purified by Ni^2+^-NTA affinity chromatography. For both purifications, similar procedures were followed as described before [12], [32].

MBP-tagged MupDivIVA was purified using amylose resin as described before [9]. The purified proteins were resolved by SDS-PAGE and analyzed after staining with coomassie brilliant blue R250. The concentration of purified proteins was estimated by Bradford assay (Bio-Rad).

### *In vitro* kinase assay

*In vitro* kinase assays of PknQ or PknQ^K41M^ (1 µg each) were carried out in kinase buffer (20 mM HEPES [pH 7.2], 10 mM MgCl_2_ and 10 mM MnCl_2_) containing 2 µCi [γ-^32^P]ATP (BRIT, Hyderabad, India) followed by incubation at 25°C for 0–30 minutes, as described previously [30], [33]. In all the reactions, kinase domain of PknQ was used (PknQ_kd_), unless specified. Myelin Basic protein (MyBP) (5 µg) was used as an artificial substrate for PknQ in a time-dependent *in vitro* kinase assay. Substrates MupFHA and MupDivIVA (5 µg each) were added with PknQ (1 µg) for phosphotransfer reactions carried out in kinase buffer containing 2 µCi [γ-^32^P]ATP followed by incubation at 25°C for 30 minutes. To determine the ionic requirements of PknQ, *in vitro* kinase assays were performed in the kinase buffer containing 20 mM HEPES [pH 7.2] with various concentrations of divalent cations (MnCl_2_, MgCl_2_, ZnCl_2_, FeCl_2_ and ammonium [iron-III] citrate) were included additionally, as indicated. Inhibition assays with ammonium [iron-III] citrate and hemin (Sigma) were performed in the presence of 10 mM Mn^2+^ and Mg^2+^ each. Reactions were terminated by 5× Laemmli sample buffer followed by boiling at 100°C for 5 minutes. Proteins were resolved by 10% SDS-PAGE and analyzed by Personal Molecular Imager (PMI, Bio-Rad). Quantification of radioactive bands was done by Quantity One 1-D Analysis Software (Bio-Rad).

### Phosphoamino-acid analysis (PAA)

Autophosphorylated ^32^P-PknQ_kd_ was separated by SDS-PAGE after *in vitro* phosphorylation and electroblotted onto Immobilon PVDF membrane (Millipore). PAA analysis by two-dimensional thin layer electrophoresis (2D-TLE) was performed as described earlier [34]. Substrates ^32^P-MupFHA and ^32^P-MupDivIVA (phosphorylated by PknQ_kd_) were analyzed similarly.

### Identification of phosphorylation sites

In order to identify the sites of autophosphorylation in PknQ, His_6_-PknQ_kd_ (5 µg) was autophosphorylated *in vitro* using 1 mM cold ATP, in the presence or absence of MupFHA (20 µg). For identification of phosphosites in MupFHA and MupDivIVA, kinase assays were performed with GST-MupFHA (5 µg) and MBP-MupDivIVA (5 µg) in the presence of 1 mM cold ATP and PknQ_kd_ (2 µg). Samples were resolved on 10% SDS-PAGE and gels were stained with coomassie brilliant blue stain. The stained bands corresponding to desired protein sizes were cut-out and used for mass-spectrometry as described earlier [35].

### Immunoblotting

Proteins were resolved by SDS-PAGE and transferred onto nitrocellulose membrane. Membrane was then blocked with 3% bovine serum albumin (Sigma) in phosphate-buffered saline containing 0.1% Tween-20 (PBST) overnight at 4°C. After blocking, the blot was washed thrice with PBST followed by incubation with primary antibodies at 1∶10,000 dilution for 1 h at room temperature. Subsequently, after five washes, the blot was incubated in secondary antibodies (Bangalore Genei) at 1∶10,000 dilution for 1 h at room temperature. The blots were developed using SuperSignal West Pico Chemiluminescent Substrate kit (Pierce Protein Research Products) according to manufacturer's instructions.

### Affinity pull-down assays

For affinity pull-down assays, Pierce Pull-Down PolyHis Protein∶Protein Interaction Kit was used (Pierce, Thermo Scientific). Briefly, His_6_-PknQ was over-expressed in *E. coli* BL-21 (DE3) cells and whole cell lysate was allowed to bind to the resin. The resin was washed to remove the non-specific unbound proteins. The lysates of *E. coli* BL-21 (DE3) cells were prepared separately containing over-expressed GST-tagged MupFHA^wt^ (wild type), MupFHA^R41A^ and MupFHA^S55A^. These three lysates were incubated with immobilized His_6_-PknQ for 1 h at 4°C. The resin was washed again followed by elution. The eluted fractions were analyzed by SDS-PAGE and immunoblotted with anti-GST antibodies (Abcam).

### Co-expression of MupFHA and MupDivIVA with PknQ

*mup018c* cloned in pGEX-5X-3 or *mup012c* cloned in pMAL-c2x were co-expressed in *E. coli* BL-21 (DE3) cells with pACYC-PknQ. PknQ^K41M^ was used as a negative control to generate unphosphorylated proteins. MupDivIVA or MupFHA were thus purified as MBP-tagged and GST-tagged proteins, respectively. Phosphorylation status of these proteins was analyzed by Pro-Q Diamond phospho-protein gel staining of SDS-PAGE gels followed by SYPRO Ruby protein gel stain, as described before [30]. These proteins were utilized for subsequent assays.

### Enzyme-linked immunosorbent assay (ELISA)

Sandwich ELISA was performed as described earlier [18]. Briefly, His_6_-tagged kinase (PknQ and its mutants or PrkD/PrkD^S162A^ mutant) or MBP-MupDivIVA (and its mutants) were dissolved in coating buffer (carbonate-bicarbonate buffer [pH 9.2]) at a concentration of 10 µg/ml and adsorbed (1 µg/well) on the surface of a 96-well ELISA plate (Maxisorb, Nunc) for 2 h at room temperature. After rinsing the wells five times with PBST, the reactive sites were blocked (3% BSA in PBST) overnight at 4°C. The adsorbed proteins were challenged with varying concentrations of soluble GST-tagged proteins (MupFHA and its mutants or Rv0020c) dissolved in blocking buffer for 1 h at room temperature. After five washes with PBST, the wells were treated with HRP-conjugated monoclonal antibody against GST (Abcam) at 1∶10,000 dilution for 1 h at room temperature. Followed by five washes, the chromogenic substrate *o*-phenylenediamine dihydrochloride (0.4 mg/ml OPD in 0.1 M phosphate/citrate buffer, pH 5.0) and H_2_O_2_ were used to measure the interaction. After addition of stop solution (2.5 M H_2_SO_4_) the absorbance was read at 490 nm. The experiments were performed 3 times with freshly purified proteins along with their mutants.

For interaction study with peptides, GST-tagged MupFHA or MupFHA^S55A^ (150 nM each) was adsorbed on the 96-well ELISA plate. After washing, 100 nM of pThr (KRpTIRR, Millipore), pSer (RRApSVA, Millipore) or a random unphosphorylated peptide (DRRRRGSRPSGAERRRRRAAAA, [36]) was allowed to interact with MupFHA. The wells were washed with PBST and His_6_-tagged PknQ (500 nM) was added. The interaction was measured as described above except the use of anti-His antibody (Abcam) at 1∶10,000 dilution. The resulting values were normalized to MupFHA^S55A^ interaction, which was used as a negative control.

## Results

### *In silico* analysis of *M. ulcerans* STPKs

**Figure 1:**
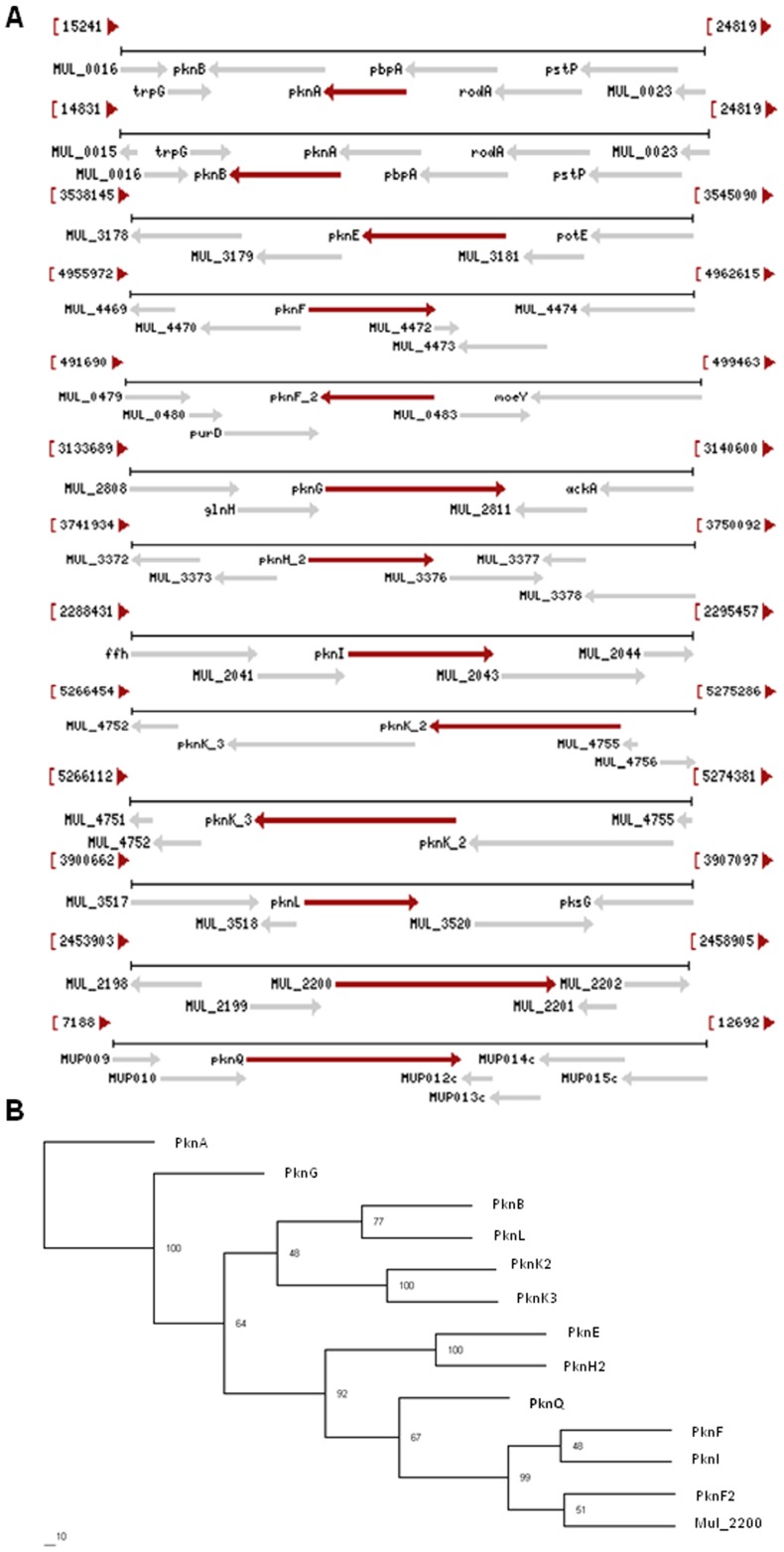
*In silico* analysis of *M. ulcerans* STPKs. (**A**) Genomic alignment of *M. ulcerans* STPKs showing conserved patterns (NCBI). Genetic patterns show twelve STPKs present in the chromosome in addition to one STPK, PknQ, which is encoded by the virulence-associated plasmid pMUM001. (**B**) Phylogenetic analysis of all *M. ulcerans* STPKs. The phylogenetic tree was generated using protein FASTA sequences of *M. ulcerans* STPKs in Phylip. PknQ clearly belongs to the PknF/PknI/Mul_2200 clade.

To identify the total number of STPKs present in *M. ulcerans*, we performed a BLASTp search using the sequence of the catalytic domain of *M. tuberculosis* PknB (1–331 aa), corresponding to the most conserved mycobacterial STPK [37]. Using this approach we identified 13 distinct ORFs encoding for STPKs, of which twelve are encoded on the chromosome and one (Mup011 or PknQ) is encoded on the virulence-associated plasmid pMUM001 (Fig. 1A). Analysis of the *M. ulcerans* STPKs showed that they possess unique kinase modules that are divergent from its close homolog, *M. marinum*. Out of the 24 putative STPK encoding genes present in *M. marinum*, only 13 are retained in *M. ulcerans*. This finding suggests that although *M. ulcerans* has evolved from *M. marinum*, it has retained only those STPKs that are necessary for its survival in humans while excluding those that may confer adaptation to *M. marinum* in fish. STPKs are broadly classified on the basis of conserved catalytic Arg/Asp (RD) residues present between subdomains VIa and VIb [30], [38]. Twelve STPKs in *M. ulcerans* belong to the RD kinase family, with PknG being the only non-RD kinase. The domain architecture of these kinases show a modular organization in which the kinase domain is located at the N-terminus (Fig. S1). SMART domain analysis revealed that 9 out of 13 kinases possess a transmembrane region that divides the N-terminal kinase domain from the C-terminal residues (Fig. S1). Multiple sequence alignments and phylogenetic analyses of *M. ulcerans* STPK sequences revealed that they belong to diverse origins and form distinct clades with strong conservation patterns in the catalytic domains, similar to *M. tuberculosis* [39] (Fig. 1B). The most striking feature of *M. ulcerans* signaling is the presence of PknQ on the virulence-associated plasmid pMUM001, which was most likely acquired for adaptability. Therefore, we decided to characterize structural features and biochemical properties of PknQ.

### Structural and biochemical characterization of PknQ

**Figure 2:**
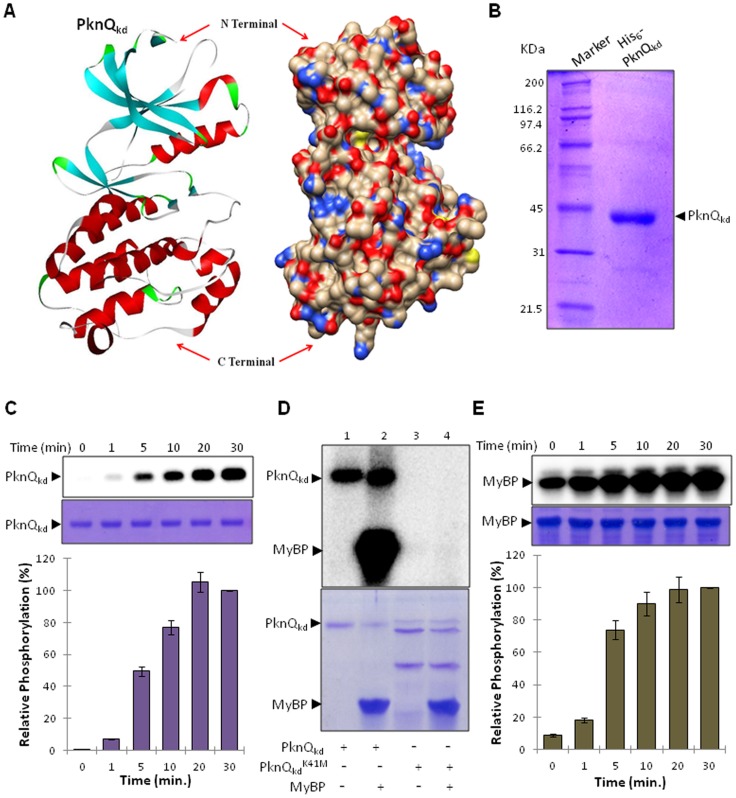
Characterization of PknQ phosphorylation. (**A**) Homology modeling showing the structure of the PknQ kinase domain (PknQ_kd_). The structure shows a ribbon diagram (left) and surface-filled model (right) of PknQ_kd_, highlighting the N- and C-terminal regions. (**B**) SDS-PAGE analysis of His_6_-tagged PknQ_kd_. PknQ_kd_ was purified to near homogeneity as observed on SDS-PAGE. (**C**) Time-dependent autophosphorylation of PknQ. PknQ was allowed to phosphorylate itself for 0–30 minutes. Relative phosphorylation (normalized to protein amounts) is plotted and corresponding autoradiogram (top) and SDS-PAGE images (bottom) are shown. The intensity of phosphorylation on protein bands was calculated using Personal Molecular Imager (Bio-Rad) using quantification software Quantity One (Bio-Rad). The phosphorylation after 30 min was taken as 100% (signal saturation) and relative phosphorylation was calculated. The experiment was repeated thrice and error bars show S.D. of three values. (**D**) Autophosphorylation of PknQ_kd_ and phosphorylation of Myelin basic protein (MyBP). Autoradiogram (top) and corresponding SDS-PAGE images (bottom) are shown. No phosphorylation was observed in the presence of the kinase-dead mutant PknQ^K41M^. (**E**) Time-dependent phosphorylation of MyBP using PknQ_kd_. Relative phosphorylation (normalized to protein amounts) is plotted and corresponding autoradiogram (top) and SDS-PAGE images (bottom) are shown. The quantification of phosphorylation intensity was done as described earlier (Fig. 1C). The experiment was repeated three times and error bars show S.D. of three values.

PknQ contains 660 amino acids with an estimated isoelectric point of 6.46. The domain analysis indicates that the cytosolic N-terminal region possesses the characteristic Ser/Thr kinase domain (kd), which is separated from the extracellular C-terminal domain harboring a FepB-like iron transporter module through a transmembrane region (Fig. S1). PknQ_kd_ (1–344 aa) harbors all 12 conserved Hank's subdomains present in eukaryotic STPK counterparts [40] (Fig. S2). Homology modeling of PknQ_kd_ and subsequent structural analysis revealed that the kinase domain consists of two lobes joined by a hinge segment. Catalysis occurs at the interface of the two lobes, where the catalytic amino acid residues interact with both ATP and the protein substrate (Fig. 2A). Based on our analysis of homology modeling and sequence similarities, we identified the residues Lys^41^ and Asp^134^ important for the phosphorylation reaction.

Therefore, in order to characterize PknQ and decipher its regulation, the gene coding for PknQ_kd_ (1–344 aa) was cloned, over-expressed and the His_6_-tag fusion protein was purified from *E. coli* (Fig. 2B). The kinase activity of purified protein was assessed by *in vitro* kinase assay, in a time-dependent manner (Fig. 2C). As shown, the maximum activity was achieved in 30 minutes under given conditions. In order to confirm PknQ phosphorylation, the conserved Lys^41^ residue was mutated to methionine as a control. The kinase and its Lys^41^ mutant were then assessed for autophosphorylation and phosphorylation of the universal substrate, myelin basic protein (MyBP). As shown in Fig. 2D, PknQ was able to phosphorylate MyBP, while PknQ^K41M^ was inactive. The phospho-transfer potential of PknQ was also assessed in a time dependent manner and phosphorylation of MyBP was quantified (Fig. 2E). This confirmed the time-dependent increase of the phospho-transfer potential of PknQ, with the saturation of signal observed after 30 min.

### Ionic requirements of PknQ

**Figure 3:**
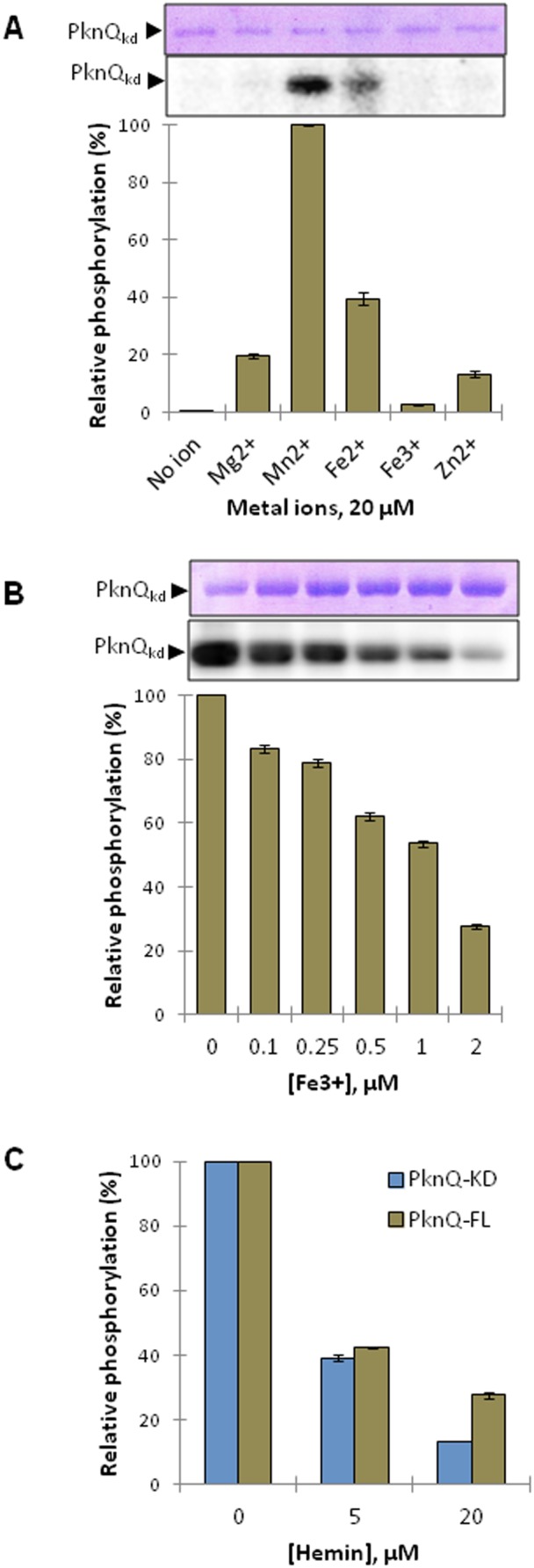
Ionic requirement of PknQ. (**A**) Ionic requirement of PknQ_kd_ using divalent cations (Mg^2+^, Mn^2+^, Fe^2+^, and Zn^2+^) and ammonium [iron-III] citrate. Maximum activity (taken as 100%) was found in the presence of Mn^2+^ and relative phosphorylation was calculated in all other lanes. The quantification of phosphorylation intensity was done as described earlier (Fig. 1C). The experiment was repeated three times and error bars show standard deviation (S.D.) of three values. Representative gel images are shown above the histogram, with the coomassie-stained gel (above panel) showing equal loading of PknQ. The lower panel is the corresponding autoradiogram. (**B**) Histogram showing inhibition of PknQ_kd_ by ammonium [iron-III] citrate (in the presence of Mn^2+^ and Mg^2+^). The quantification of phosphorylation intensity was done as described earlier (Fig. 1C). Maximum activity was found in the absence of ammonium [iron-III] citrate and was taken as 100%. Relative phosphorylation was calculated in all other lanes. The experiment was repeated three times and error bars show S.D. of three values. Representative gel images shown above indicate equal loading of PknQ_kd_ (above panel) and the corresponding autoradiogram (lower panel). (**C**) Histogram showing inhibition of full-length PknQ (PknQ_fl_) and kinase domain (PknQ_kd_) by hemin (in the presence of Mn^2+^ and Mg^2+^). Maximum activity was found in the absence of hemin and was taken as 100%. Relative phosphorylation was calculated in all other lanes. The experiment was repeated three times and error bars show S.D. of three values.

The domain organization of PknQ includes a C-terminal ion transporter module, indicating that cofactors may play an important role in regulating its activity. To analyze the PknQ ionic requirements, *in vitro* kinase assays were performed with [γ-^32^P]ATP in the presence of different divalent cations known to regulate the activity of STPKs [32], [33], [41]. PknQ kinase activity was found to be dependent on the presence of Mn^2+^, although slight activation was also observed in the presence of Mg^2+^, Fe^2+^, and Zn^2+^ ions (Fig. 3A). However, no activation was observed in the presence of Fe^3+^ ions, rather they inhibited the activity of PknQ in a concentration-dependent manner, even in the presence of Mn^2+^ and Mg^2+^ (Fig. 3B). To confirm these results we performed an *in vitro* kinase assay with PknQ_kd_ and hemin, which contains integrated Fe^3+^ ions. Since the C-terminal FepB domain of PknQ is known to release iron from heme [42], the kinase activity of PknQ_fl_ was also checked. Hemin indeed inhibited the activity of PknQ_fl_ as well as PknQ_kd_ (Fig. 3C).

### Identification of PknQ phosphorylation sites

**Figure 4:**
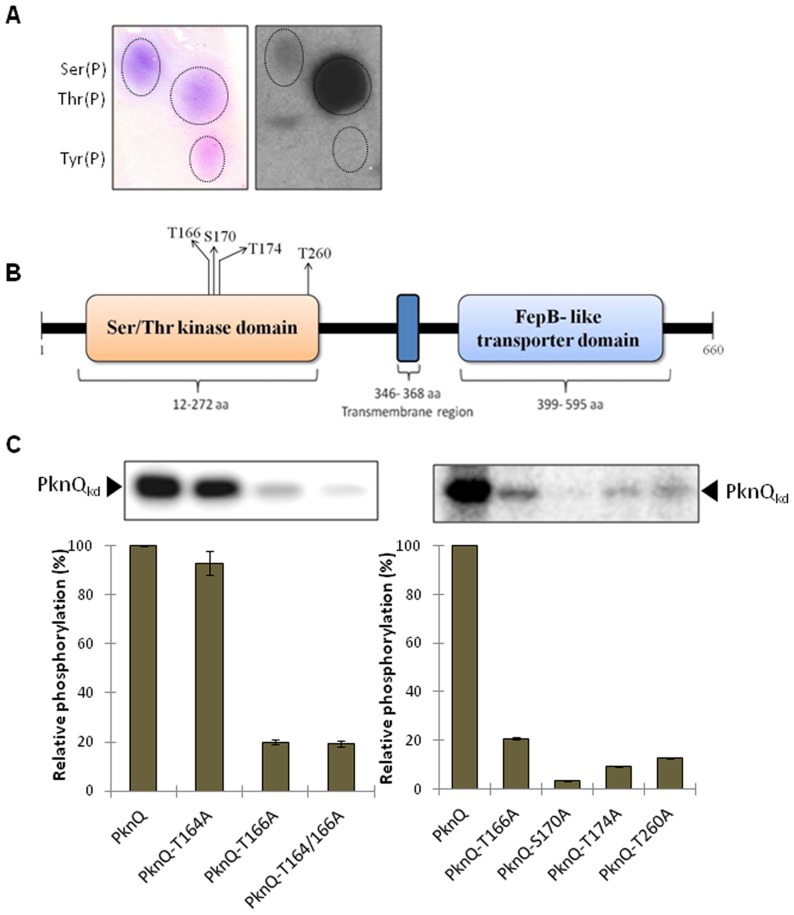
Phosphorylation sites of PknQ. (**A**) Phosphoamino acid (PAA) analysis of PknQ_kd_ autophosphorylation using 2D-TLE. The left panel shows ninhydrin-stained phospho-amino acid spots and the right panel shows the corresponding autoradiogram. Phosphorylation was detected on the spots corresponding to Ser(P) and Thr(P). (**B**) Domain architecture of PknQ showing catalytic domain (cytosolic) and extracellular FepB-like transporter domain. Four of the phosphorylated residues that are present in the activation loop are marked. (**C**) Single and double site mutants of PknQ were generated for the two most conserved activation loop residues (Thr^164^ and Thr^166^) (left histogram). Single site mutants of PknQ were subsequently generated for multiple residues (Ser^170^, Thr^174^ and Thr^260^) as identified by mass spectrometry (right histogram). Phosphorylation of PknQ was taken as 100% and relative phosphorylation was estimated. The experiment was repeated three times and error bars show S.D. of three values. A representative autoradiogram is shown above each histogram. PknQ^S170A^ exhibited the maximum loss in phosphorylation.

We identified the phosphorylated amino acid(s) on PknQ_kd_ using 2D-TLE. We found that both serine and threonine residues were phosphorylated, while no phosphorylation was observed on tyrosine residues (Fig. 4A). Mass spectrometric analysis of PknQ_kd_ identified multiple phosphorylated Ser/Thr residues (Table 1, Fig. 4B). Sequence analysis of residues proximal to the phosphosites identified proline residues in close proximity to seven phosphosites. The importance of proline in proximity to phospho-acceptor residues has been well established in eukaryotic STPKs [43], [44]. Thus, these residues may help in PknQ autophosphorylation and would be useful for identifying PknQ-specific phosphorylation motifs.

**Table 1:** Phosphorylated residues of PknQ identified by *in vitro* kinase assays.

Phosphorylated tryptic peptide sequence of autophosphorylated PknQ	Phosphorylated residue(s)	Number of detected phosphate groups LC/MS/MS
HP**pT**LPRSDALK [31–41]	**T33**	1
HP**pT**LPR**pS**DALK [31–41]	**T33+S37**	2
IL**pS**AELSQDEQFR [42–54]	**S44**	1
ILSAEL**pS**QDEQFR [42–54]	**S48**	1
EADLAA**pT**LSHPNIVTVFNR [60–78]	**T66**	1
EADLAA**pT**LpSHPNIVTVFNR [60–78]	**T66+S68**	2
LHA**pT**VLTPAR [100–110]	**T104**	1
VAAII**pT**DVGAALDYAHSR [110–128]	**T116**	1
DIKP**pS**NFLVSADHER [134–148]	**S138**	1
DIKPSNFLV**pS**ADHER [134–148]	**S143**	1
AFDDT**pT**LTAIGSLVGTASYAAPEAIQGGSVDQR [159–191]	**T164**	1
AFDDT**pT**L**pT**AIGSLVGTASYAAPEAIQGGSVDQR [159–191]	**T164+T166**	2
AFDDT**pT**L**pT**AIG**S**LVG**T**ASYAAPEAIQGGSVDQR [159–191]	**T164+T166+(S170 or T174)**	3
IGSLVG**pT**ASYAAPEAIQGGSVDQR [168–191]	**T174**	1
FP**pT**AGALAGAAR [258–269]	**T260**	1
AAL**pS**GQPLPQAPPGGPK**pT**R [270–288]	**S273+T287**	2
AALSGQPLPQAPPGGPK**pT**R [270–288]	**T287**	1
IWAAPPLSYP**pT**TRPPGI [289–305]	**T299**	1
GFAGAAHPGLAGAA**pS**SSDER [314–333]	**S328**	1
AGAAHPGLAGAAS**pS**SDER [316–333]	**S329**	1

Sequences of the phosphorylated peptides identified in autophosphorylated PknQ in the absence of MupFHA are indicated, as determined by mass spectrometry. Phosphorylated residues (pT or pS) are shown in bold.

We next generated the two phospho-ablative mutants of Thr^164^ and Thr^166^ sites, since their homologous residues are known to be the most conserved phosphorylated residues in the activation loop of STPKs [33], [45], [46]. Equal amounts of PknQ and its mutants were used in the *in vitro* kinase assays with [γ-^32^P]ATP, resolved on SDS-PAGE, and analyzed by autoradiography. In the quantitative analysis, there was no significant loss in phosphorylation observed in the PknQ^T164A^ single mutant, while PknQ^T166A^ and PknQ^T164/166A^ showed significant loss in signal intensity (Fig. 4C). The Ser^170^ and Thr^174^ residues were also present in the activation loop of the PknQ catalytic domain (Fig. 4B). In order to determine the impact of phosphorylation of these residues together with the residues of the juxtamembrane region, site-directed mutagenesis was performed to generate mutations at these sites. We observed that the PknQ^S170A^ mutant had a marked reduction in phosphorylation activity, suggesting that this site is critical for PknQ activation (Fig. 4C). PknQ^T174A^ and PknQ^T260A^ mutants also exhibited ∼80% loss in phosphorylation signal. Comparison of PknQ, PknQ^K41M^ and PknQ^T174A^ phosphorylation indicated that PknQ^T174A^ is partially active and is distinct from PknQ^K41M^ derivative, which is completely inactive (Fig. S3A). No loss in phosphorylation levels was observed with other single site mutants (Fig. S3B), indicating that phosphorylation of these residues (including the juxtamembrane region) does not seem to play a major role in PknQ autokinase activity. Hence, the most important phosphorylation sites in the PknQ are Ser^170^, Thr^174^, Thr^166^ and Thr^260^. In most bacterial STPKs, activity is regulated by threonine residues in the activation loop and not by serine [11], [30]. Thus, it was surprising that Ser^170^ plays a major role in PknQ activation and indicates a novel feature of PknQ in the mycobacterial kinome.

### Identification of PknQ interacting proteins in *M. ulcerans*

In order to regulate and amplify signals, protein kinases associate and interact with a number of proteins within the cell. Most of the bacterial protein kinases are known to exhibit synteny with their substrates. In *M. ulcerans*, analysis of *pknQ* genetic loci revealed a possible operon between *mup012c* and *mup018c*. These genes code for proteins whose homologs are known as key kinase substrates in *M. tuberculosis* and many other bacteria. The search for kinase interacting domain took us to the FHA domain that is present in Mup018c (renamed as MupFHA). In *M. tuberculosis*, several STPK-FHA domain containing protein partners are broadly conserved at the same genetic loci, such as PknB-Rv0019c, PknF-Rv1747 or PknH-EmbR [18], [47], [48].

FHA domains are comprised of approximately 55–75 amino acids with three highly conserved blocks- GR, SXXH, and NG- separated by divergent spacer regions [49]. Structurally, the FHA domain contains an 11-stranded β-sandwich with small helical insertions at the loops connecting the strands [49]. Recent reports suggest that the FHA domain-containing proteins interact with and recruit other phospho-proteins [17], [18], [21]. In eukaryotes, STPKs associate with multiple signaling domains, such as the BRCT, 14-3-3, Polo box, SH2, WW, WD40, and FHA domains [18], [50], while in bacteria, only FHA domains have been identified as the conserved STPK interacting domains. In addition, their role has been studied in important cellular processes [49]. In order to determine the status of FHA domain containing proteins in *M. ulcerans*, we performed a BLASTp search using Rv0020c of *M. tuberculosis* as a query [51] and found six additional FHA domain containing proteins (compared to *M. marinum*, which has ten FHA domain containing proteins, including the one encoded by the pMUM003 plasmid in *M. marinum* DL240490). A domain analysis and homology search showed that all of the proteins have functional homologs in *M. tuberculosis* except MupFHA (Table 2). To further explain the relationships of MupFHA with other FHA domain homologs in other bacteria that carry a megaplasmid, for example non-pathogenic bacteria *Mesorhizobium cicero*, we performed BLASTp search with MupFHA sequence in the *M. cicero* database. There are two FHA domain containing proteins encoded by the *M. cicero* megaplasmid. However, the two proteins named Mesci_6382 and Mesci_6368 are annotated as type-VI secretion system FHA domain proteins and thus, are different from MupFHA. Similarly, in another pathogenic bacterium, *Yersinia pestis*, the virulence-associated plasmid pLB1 encodes an FHA domain protein YscD that is a part of the type-III secretion apparatus. Thus, it is possible that MupFHA, YscD, Mesci_6382 and Mesci_6368 are related evolutionarily. However, MupFHA does not contain any such secretory domain indicating divergence at some point. Thus, these analyses indicate that the MupFHA is a unique FHA domain containing protein present in the virulence-associated plasmid of *M. ulcerans*.

**Table 2:** Conserved FHA-domains in *M. ulcerans*.

*M. ulcerans*	*M. tuberculosis*
MUL_1424	Rv1747
MUL_0024	Rv0020c
MUL_0023	Rv0019c
MUL_3149	Rv1747
MUL_4018	EmbR, Rv1267c
MUL_3046	GarA, Rv1827
MUP018c	Rv3863

### Structural analysis of PknQ and MupFHA domain interactions through docking

**Figure 5:**
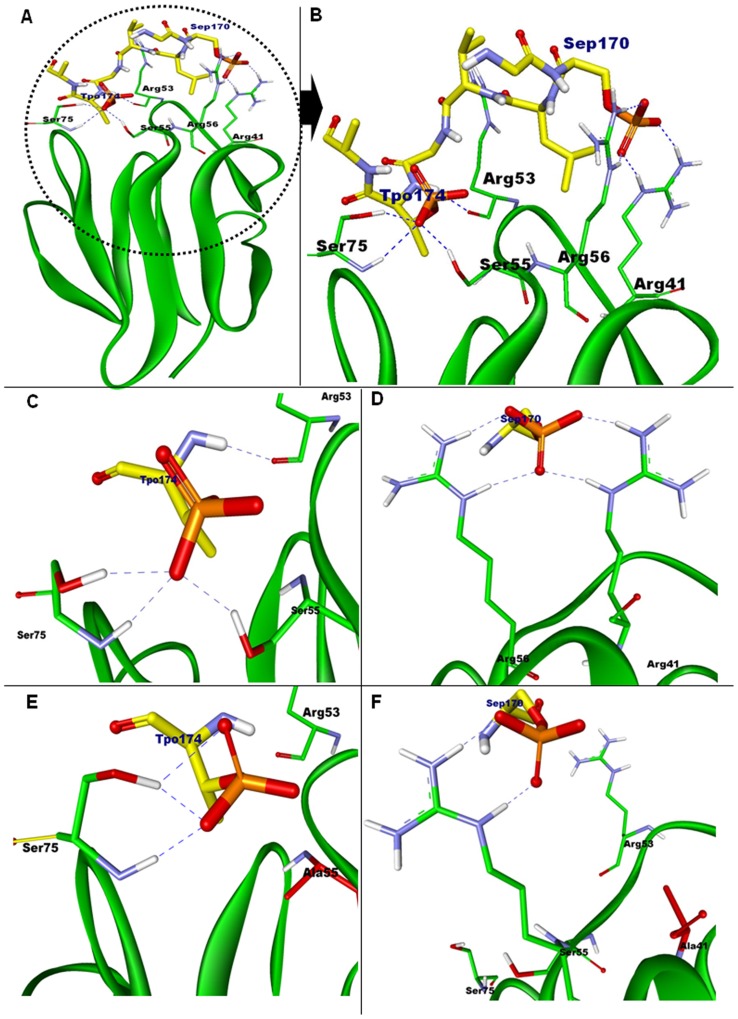
Docking analysis of PknQ with MupFHA domain. (**A**) Homology modeling derived structural models showing docking of wild-type PknQ (stick diagram) with the wild-type FHA domain variable loop region of MupFHA (green ribbon diagram). Phosphate group (orange) has been added to Ser^170^ and Thr^174^ of PknQ and the phosphorylated residues have been renamed as Sep170 and Tpo174, respectively. The red encircled region of interaction has been enlarged in (**B**). The residues Arg^41^, Arg^53^, Ser^55^, Arg^56^ and Ser^75^ of MupFHA show stable interactions with the PknQ activation loop and form H-bonds with the negatively charged pSer^170^ (Sep170) and pThr^174^ (Tpo174). (**C**) Enlarged region of interaction between PknQ-pThr^174^ and MupFHA. Canonical interaction of pThr^174^ is observed showing H-bonds with Arg^53^, Ser^55^ and Ser^75^ of MupFHA (see text). (**D**) Enlarged region of interaction between PknQ-pSer^170^ and MupFHA. PknQ-pSer^170^ is shown to be anchored by the residues Arg^41^ and Arg^56^ of MupFHA. (**E**) Region of interaction between PknQ-pThr^174^ and MupFHA^S55A^ (in red stick). (**F**) Region of interaction between PknQ-pSer^170^ and MupFHA^R41A^ (in red stick). Both (E) and (F) show the loss of H-bond network and thus destabilized interaction between PknQ and MupFHA.

*mup018c* (*mupFHA*) is present in the vicinity of *pknQ* (*mup011*), suggesting that the two proteins encoded by these genes may interact with each other. In order to confirm this hypothesis, we first performed multiple sequence alignment of MupFHA and other known FHA-domain containing proteins. The alignment shows that the five most conserved residues of FHA-domain (Gly^40^, Arg^41^, Ser^55^, His^58^ and Asn^76^) are also present in MupFHA (Fig. S4). Next, we generated a structural model of the FHA domain of MupFHA using *M. tuberculosis* Rv0020c as a template. MupFHA was found to contain 11 β-strands as is true for all the FHA-domain containing proteins [24], [49]. PknQ_kd_ and MupFHA models were then used for the docking studies and to characterize the key interacting residues. Amongst the major phosphorylation sites of PknQ (Thr^166^, Thr^174^, Ser^170^ and Thr^260^), Thr^166^ and Thr^260^ do not make close contact with MupFHA (Fig. S5). Thus, the docking was further performed using PknQ phosphorylated at Ser^170^ and Thr^174^. PknQ was found to interact with the residues present in the loops β3–β4, β4–β5 and β6–β7 of MupFHA, as described previously for other FHA domains [24], [52], [53]. Figure 5 shows the various docked complexes emphasizing the role of activation loop residues PknQ-pSer^170^ (Sep170) and PknQ-pThr^174^ (Tpo174) in stabilizing the complex formed with MupFHA (Fig. 5A and 5B). PknQ-Thr^174^ exhibits canonical binding with the FHA-domain residues as observed in various previous studies [24], [52], [53]; and forms H-bonds with Ser^55^, Ser^75^ and Arg^53^ residues of MupFHA (Fig. 5B and 5C). PknQ-pThr^174^ interacts with MupFHA-Ser^55^, one of the five most conserved FHA-domain residues, exactly in the same manner as Human Rad53-FHA1 and *M. tuberculosis* Rv0020c bind to their phospho-peptides [24], [53]. Rv0020c contains a Thr at position corresponding to MupFHA-Ser^75^ (Fig. S4) that also forms H-bond with pThr of the phospho-peptide [24]. The MupFHA model differs from the known Human Rad53 structure with respect to the binding of pThr^174^ with the other absolutely conserved residue MupFHA-Arg^41^. MupFHA-Arg^41^ was not found to interact with pThr^174^; instead it forms H-bond with PknQ-pSer^170^. MupFHA-Arg^41^ holds PknQ-pSer^170^ like a clip with the help of MupFHA-Arg^56^ (Fig. 5B and 5D), reminiscent of pSer binding by Human PNK-FHA and *M. tuberculosis* Rv0020c [24], [52]. Presence of negatively charged pSer residue in proximity to these positively charged arginine residues have been proposed to provide favorable interaction [52]. MupFHA-Arg^56^ is not among the five absolutely conserved residues but is found in most of the FHA domains (Fig. S4). Rad53-FHA1 contains an asparagine at this site which also forms H-bond with pThr [53]. This analysis indicates that residues Ser^170^ and Thr^174^ of PknQ are important for its interaction with MupFHA.

To further apprehend the interaction between these two proteins, we performed docking of the FHA domain mutants of the two absolutely conserved residues- Arg^41^ and Ser^55^ with the wild-type PknQ_kd_. Significant loss of H-bonds within PknQ activation loop was observed in both the cases (Fig. 5E and 5F). Serine to alanine substitution at position 55 of MupFHA led to the loss of H-bonding with PknQ-Thr^174^ (Fig. 5E). Similarly, MupFHA^R41A^ mutation abolished the formation of H-bonds with PknQ-Ser^170^ that were essential for holding the complex (Fig. 5F). Together, these analyses identified critical amino acid residues involved in the PknQ and MupFHA interaction.

### Structural basis of MupFHA-pSer/pThr interaction

**Figure 6:**
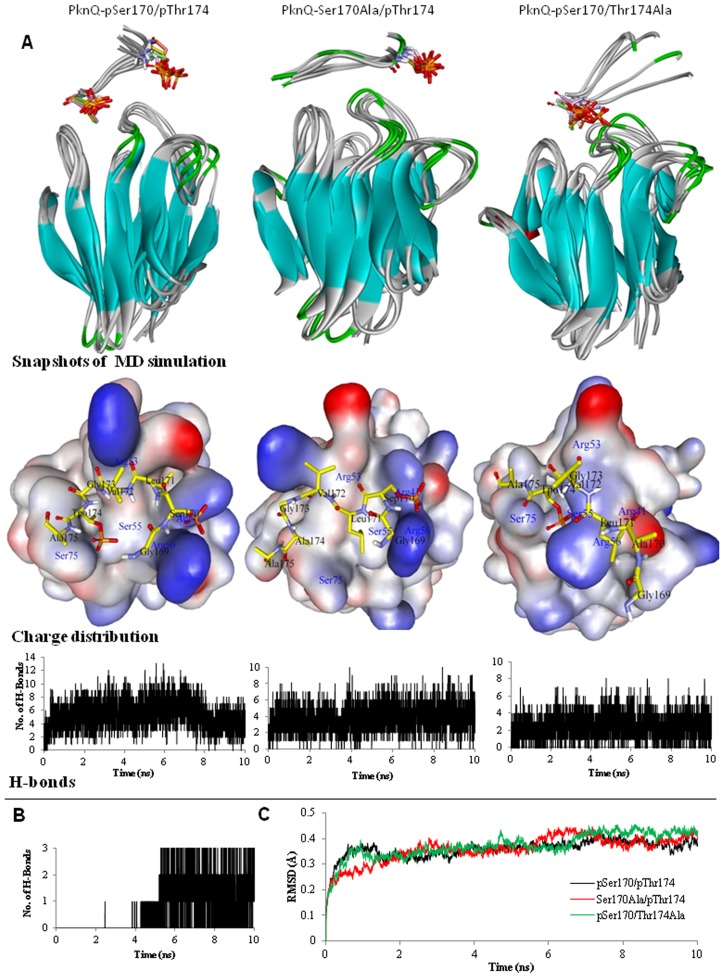
Molecular dynamics simulation of PknQ and MupFHA. (**A**) MD simulation analysis of interaction between MupFHA and PknQ-pSer170/pThr174, PknQ-pSer170/Thr174Ala or PknQ-Ser170Ala/pThr174. The upper panel shows snapshots taken every 2 ns to assess the backbone fluctuations of the complex of MupFHA (ribbon representation) with PknQ activation loop (stick representation). The dually phosphorylated activation loop of PknQ shows the most significant interaction and a stable complex. The middle panel shows the charge distribution during the interaction of MupFHA with PknQ. The central part of the PknQ activation loop is shown to interact with the FHA domain surface. The charge distribution along with the molecular surface of FHA is shown coloured according to the electrostatic potential- blue for positive and red for negative. Phosphate groups added to Ser^170^ and Thr^174^ are shown in orange color. The lower panel shows H-bonding between all the complexes during the entire simulation as a function of time. (**B**) H-bond plot for PknQ-pThr^174^ in PknQ-Ser170Ala/pThr174 and MupFHA-Arg^56^. Residue pThr^174^ elucidates the canonical interaction after 4 ns which was occupied by pSer^170^ in dually phosphorylated activation loop. (**C**) RMSD curve for a total of 10 ns during the MD simulation.

Molecular dynamics simulation was performed to probe the conformational relaxation of the PknQ-MupFHA complex and to evaluate the dynamic interaction of phosphorylated residues in PknQ activation loop (pSer^170^ and pThr^174^) with the FHA domain (Supplementary movie files S1, S2 and S3). Apart from the dually phosphorylated PknQ-pSer170/pThr174 (Fig. 6A, left panel), the molecular dynamics simulation was also performed with PknQ-pSer170/Thr174Ala (Fig. 6A, middle panel) or PknQ-Ser170Ala/pThr174 (Fig. 6A, right panel). The snapshots of complexes at different time points suggest that the dually phosphorylated form of PknQ makes the most stable complex with MupFHA. Both the mutations- T174A and S170A, lead to destabilization and fluctuations in the complex (Fig. 6A). The analysis of overall H-bonding in the complex again shows that the dually phosphorylated PknQ forms a stable complex with MupFHA. The number of H-bonds decreases in the case of complex formed with PknQ mutants.

Analysis of charge distribution in the complex suggests that in the dually phosphorylated model, Ser^55^ and Ser^75^ of MupFHA play an important role in the recognition and form 3–4 H-bonds during interaction with PknQ-pThr^174^. PknQ-pSer^170^ interacts with the Arg^41^ and Arg^56^ residues of MupFHA. These interactions are maintained even when there is a Thr^174^ to alanine substitution in PknQ. When Ser^170^ is substituted to alanine, MupFHA-Arg^56^ becomes available for H-bonding. There is a relative movement of Arg^56^ so that it now interacts with PknQ-pThr^174^. The analogous interaction of MupFHA-Arg^56^ and PknQ-pThr^174^ has also been observed previously in other studies [24], [52], but is not seen when there is a pSer^170^ residue in PknQ. Thus, MupFHA-Arg^56^ moves towards PknQ-pThr^174^ only when pSer^170^ is absent. In this case, when H-bonds between PknQ-Thr^174^ and MupFHA-Arg^56^ were analyzed, it indicated that before 4 ns there is no significant interaction, but after 4 ns, 2–3 H-bonds are formed between these two residues (Fig. 6B).

The RMSD values were then calculated and plotted for all the three complexes (Fig. 6C). The graph shows an initial structural rearrangement (1–2 ns) contributing to higher fluctuations in RMSD values for all the three protein complexes (Fig. 6C). The complexes formed with single phosphorylated residue of PknQ exhibit higher structural rearrangements and higher RMSD fluctuations compared to the double phosphorylated protein complex. These results indicate that Thr^174^ and Ser^170^ of PknQ are important mediators of the interaction with MupFHA. Phosphorylation of Ser^170^ may regulate interaction of MupFHA with PknQ by making MupFHA-Arg^56^ and Arg^41^ unavailable for pThr^174^.

### Characterization of the PknQ_kd_ and MupFHA interaction

**Figure 7:**
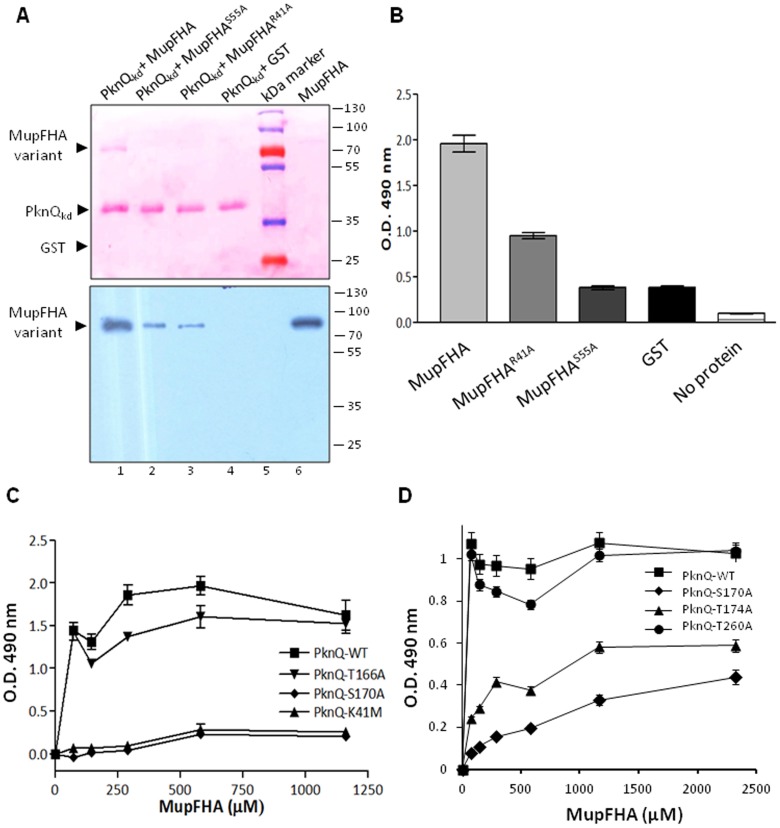
Interaction of MupFHA with PknQ. (**A**) Affinity pull-down assay of PknQ with MupFHA and its FHA domain mutants. Immobilized His_6_-PknQ was allowed to interact with GST-tagged MupFHA and its mutants (MupFHA^R41A^ and MupFHA^S55A^). GST served as a negative control for the pull-down assay and showed no interaction, which confirmed the specificity of the PknQ∶MupFHA interaction (lane 4). The eluted fractions were immunoblotted with anti-GST antibodies to assess the levels of interactions. The upper panel shows ponceau-stained membrane and lower panel is the corresponding immunoblot. As evident from the immunoblot, MupFHA was eluted together with PknQ, while MupFHA^R41A^ and MupFHA^S55A^ were weakly co-eluted. Purified MupFHA was used as a positive control for immunoblotting (lane 6). (**B**) Histogram showing ELISA-based interaction of PknQ with MupFHA and its variants. PknQ strongly interacts with wild-type MupFHA, while the interactions with MupFHA^R41A^ and MupFHA^S55A^ mutants were significantly decreased (also observed in the pull-down assay). The experiment was performed three times (error bars show standard error of three values) and GST/no protein were used as negative controls. (**C**) Graph showing ELISA-based interaction of MupFHA with PknQ and PknQ^S170A^ mutant. Loss of PknQ-Ser^170^ leads to an attenuated interaction with the MupFHA, indicating the role of this pSer residue in the interaction with MupFHA. No interaction was observed with PknQ^K41M^, while loss of PknQ-Thr^166^ did not significantly affect its interaction with MupFHA. The experiment was performed three times (error bars show S.E. of three values). (**D**) Graph showing ELISA-based interaction of MupFHA with PknQ, PknQ^S170A^, PknQ^T174A^ and PknQ^T260A^ mutants. PknQ^T174A^ mutant shows decreased interaction with MupFHA while PknQ^T260A^ exhibits comparable interaction to the MupFHA wild type protein. The experiment was performed three times (error bars show S.E. of three values).

To validate the interactions between MupFHA and PknQ observed through docking analysis, we performed affinity pull-down assays. *Mup018c* was cloned in an *E. coli* expression vector and the corresponding protein MupFHA was purified as a GST-tagged fusion protein. Site-specific mutants of MupFHA were also generated and used for pull-down assays with PknQ to validate the docking studies. Pull-down assays showed that PknQ strongly interacts with wild-type MupFHA and that the interaction was reduced with the MupFHA^R41A^ and MupFHA^S55A^ mutants (Fig. 7A).

To further validate the interaction of the FHA domain with PknQ, we studied specific protein-protein interactions through sandwich ELISA. His_6_-PknQ was adsorbed on a 96-well plate and allowed to interact with equimolar amounts of GST-tagged substrates. Significant interaction was observed between His_6_-PknQ and GST-MupFHA (Fig. 7B). Interaction assays of PknQ were also performed with the GST-MupFHA^R41A^ and GST-MupFHA^S55A^ mutants. The interactions were severely disrupted by mutating the two residues of MupFHA (Fig. 7B). Thus, our results confirmed that the interaction of PknQ∶MupFHA occurred via the FHA domain and requires Arg^41^ and Ser^55^.

### Specificity of MupFHA domain towards phospho-residues

Docking studies and MD analysis indicated the critical role of the activation loop residue Ser^170^ in the PknQ∶MupFHA interaction along with the pThr^174^. FHA domains are primarily pThr binding domains. In fact, such pThr specificity helps in decreasing the potential interaction sites of FHA domains, as 90% of all Ser/Thr kinase activity in eukaryotes is directed towards serine phosphorylation [54]. However, in prokaryotes, and even more so in *Mycobacterium*, STPKs more often act upon threonine residues [11]. However, PknQ is an exception, as it requires Ser^170^ in addition to threonine residues. Therefore, to investigate the role of Ser^170^ in PknQ∶MupFHA interaction, we performed an ELISA with MupFHA and PknQ^S170A^. Our results showed a loss of interaction between MupFHA and PknQ^S170A^ compared to wild-type PknQ and PknQ^T166A^ (Fig. 7C). The interaction of PknQ^S170A^ was similar to the kinase inactive mutant PknQ^K41M^, which is the completely unphosphorylated form of the kinase. We also compared this interaction with interaction of other key phospho-residue mutants (Thr^174^ and Thr^260^) (Fig. 7D). Apart from Ser^170^, the only residue important for interaction is Thr^174^ confirming the results obtained from structural modeling (Fig. 5A).

Furthermore, MupFHA-PknQ interaction was probed in competition ELISA-based assays using synthetic peptides phosphorylated either on threonine or serine residue. Immobilized MupFHA was first incubated with pSer, pThr or random unphosphorylated peptide and competitively replaced by PknQ. In this assay, the MupFHA-PknQ interaction was proportionally dependent on MupFHA∶phospho-peptide binding. We observed that both pThr and pSer peptides showed affinity to MupFHA but not the unphosphorylated random peptide (Fig. S6). Notably, the binding of MupFHA with both phospho-peptides was not very strong, which probably indicates the role of neighbouring residues (of phospho-acceptor site) in these interactions. To further probe the pSer/pThr interaction, we also utilized *Bacillus anthracis* kinase PrkD that autophosphorylates on Ser^162^ residue, in addition to several threonine and tyrosine residues [30]. MupFHA was allowed to bind with PrkD and PrkD^S162A^ where the interaction was found to be reduced after mutation of Ser^162^ (Fig. S7). Together, these results indicate the affinity of MupFHA with both pThr and pSer.

### Identification of MupFHA as a PknQ substrate and role of PknQ autophosphorylation sites in MupFHA phosphorylation

**Figure 8:**
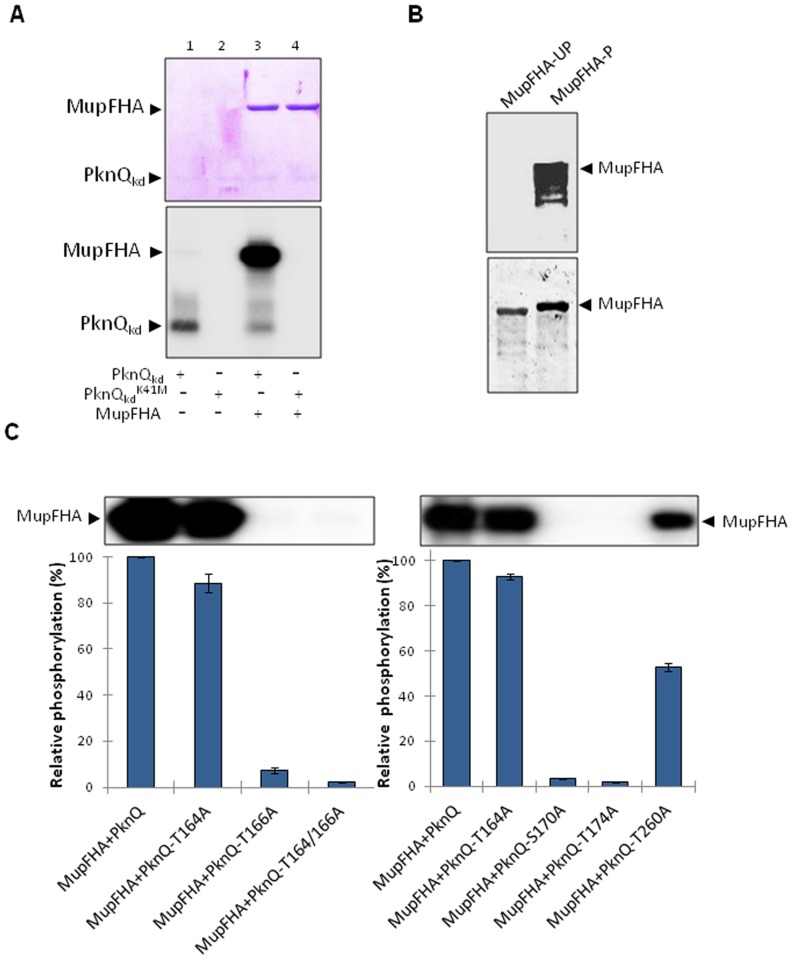
Phosphorylation of MupFHA by PknQ. (**A**) *In vitro* kinase assay showing phosphorylation of MupFHA by PknQ and PknQ^K41M^. The upper panel shows a coomassie-stained SDS-PAGE and the lower panel shows the corresponding autoradiogram. MupFHA is phosphorylated by PknQ while no phosphorylation was observed with PknQ^K41M^. Surprisingly, in the presence of MupFHA, the level of PknQ phosphorylation was reduced (lanes 1 and 3). (**B**) Phosphorylation status of MupFHA, co-expressed with PknQ or PknQ^K41M^ in *E. coli*, was estimated using ProQ Diamond phosphoprotein staining (upper panel). MupFHA co-expressed with PknQ was found to be phosphorylated (MupFHA-P), while no phosphorylation was observed when it was co-expressed with PknQ^K41M^ (MupFHA-UP). The same gel was stained with Sypro Ruby stain (lower panel) to show equal loading of both samples. (**C**) The mutants of PknQ, which showed loss in autophosphorylation potential, were used to assess their phosphotransfer ability on MupFHA. Phosphorylation by wild-type PknQ was taken as 100% and relative phosphorylation was calculated. The experiments were repeated three times and error bars show S.D. of three values. Representative autoradiograms with MupFHA bands are shown above the histograms (left and right panels).

FHA domain containing proteins are known to be phosphorylated by their neighboring kinases [18], [20], [48]. To explore this possibility in *M. ulcerans*, we performed kinase assays with PknQ and MupFHA and found that PknQ phosphorylated MupFHA, while no phosphorylation was observed with PknQ^K41M^ (Fig. 8A). To further validate MupFHA phosphorylation by PknQ, we co-expressed the two proteins in *E. coli* using compatible vectors, one expressing either PknQ or PknQ^K41M^ (pACYCDuet-1) and the second expressing MupFHA (pGEX-5X-3). Affinity purified MupFHA that was co-expressed with PknQ (renamed as MupFHA-P) showed an intense phosphorylation signal, while no phosphorylation was found in MupFHA co-expressed with PknQ^K41M^ (MupFHA-UP) (Fig. 8B).

We also assessed the role of active site residues of PknQ on its ability to phosphorylate MupFHA. Considerable loss of phosphotransfer was observed with the mutants- PknQ^T166A^, PknQ^S170A^ and PknQ^T174A^ compared to the wild-type and PknQ^T164A^ (Fig. 8C). Therefore, in addition to autophosphorylation, the residues Ser^170^, Thr^166^, and Thr^174^ are also critical for regulating the phosphotransfer.

### Identification of MupFHA phosphorylation sites and role of the FHA domain in PknQ-mediated phosphorylation of MupFHA

**Figure 9:**
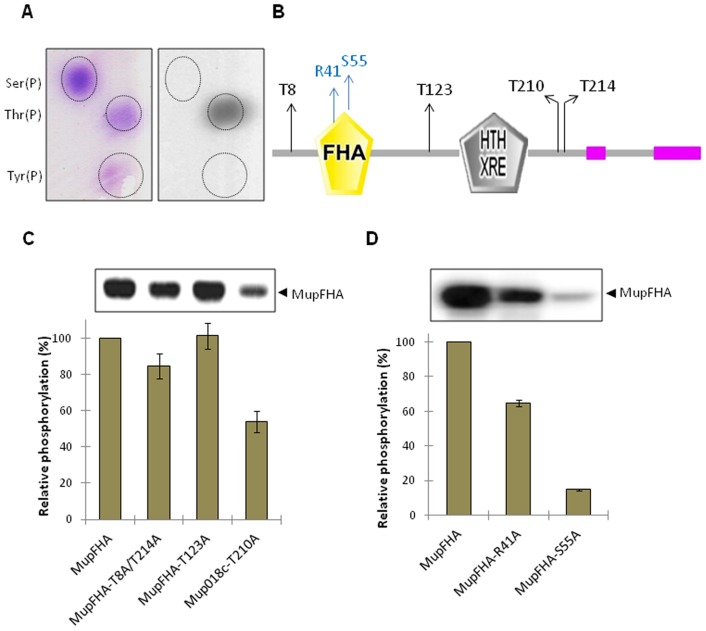
Phosphorylation sites of MupFHA and role of FHA domain residues. (**A**) PAA analysis of MupFHA phosphorylated by PknQ. The left panel shows ninhydrin-stained phosphoamino acid spots and the right panel shows the corresponding autoradiogram. Phosphorylation was detected on the spot corresponding to Thr(P). (**B**) Domain architecture of MupFHA analyzed by SMART software. The four threonine residues (Thr^8^, Thr^123^, Thr^210^, and Thr^214^) that were phosphorylated are marked. The two conserved residues in the FHA domain (Arg^41^ and Ser^55^) are also marked. (**C**) Multiple mutants of MupFHA phosphorylation sites were generated and the loss in phosphorylation by PknQ was assessed. Phosphorylation on wild-type MupFHA was taken as 100% and relative phosphorylation was calculated. As shown in the histogram, maximum loss was observed when Thr^210^ of MupFHA was mutated. The experiment was repeated three times and error bars show S.D. of three individual values. A representative autoradiogram with MupFHA bands is shown above the histogram. (**D**) Histogram showing phosphorylation of MupFHA and its FHA domain mutants by PknQ. Phosphorylation on wild-type MupFHA was taken as 100% and relative phosphorylation was calculated. A significant loss was observed for the MupFHA^S55A^ mutant compared to MupFHA^R41A^. The experiment was repeated three times and error bars show S.D. of three values. A representative autoradiogram with MupFHA bands is shown above the histogram.

To identify the MupFHA residues phosphorylated by PknQ, PAA analysis was performed. PknQ phosphorylated MupFHA on threonine residue(s), while no signal was observed on the spots corresponding to pSer and pTyr (Fig. 9A). We subsequently identified the phosphorylation sites in MupFHA by mass spectrometry. PknQ phosphorylated MupFHA on four threonine residues (Thr^8^, Thr^123^, Thr^210^, and Thr^214^), which confirmed the results obtained from the PAA analysis (Fig. 9B, Table 3). Interestingly, none of these residues were present in the FHA domain. To verify these phosphorylation sites, we generated single and multiple phospho-ablative mutants of MupFHA and compared the level of phosphorylation in all the mutants with wild-type MupFHA. Surprisingly, loss of only Thr^210^ resulted in approximately a 50% reduction in phosphorylation signal, while ∼20% loss of signal intensity was observed with the double mutant MupFHA^T8A/T214A^ (Fig. 9C). No loss was observed for the MupFHA^T123A^ mutant.

**Table 3:** Phosphorylated residues of MupFHA identified by *in vitro* kinase assays.

Phosphorylated tryptic peptide sequence of MupFHA phosphorylated by PknQ	Phosphorylated residue(s)	Number of detected phosphate groups LC/MS/MS
MQQPTEH**pT**TPMDSLAPPALVIK [1–22]	**T8**	1
**pT**EHIEDTSDPK [122–133]	**T123**	1
GHIMWLYEQDIQPDEER**pT**HVLTATTPVPEITGATK [193–227]	**T210**	1
THVL**pT**ATTPVPEITGATK [210–227]	**T214**	1

Sequences of the phosphorylated peptides identified in MupFHA phosphorylated by PknQ are indicated, as determined by mass spectrometry. Phosphorylated residues (pT) are shown in bold.

Since the FHA domain of MupFHA is involved in its interaction with PknQ, it was essential to study the role of the FHA domain in its phosphorylation. Therefore, the two conserved FHA domain residues, Arg^41^ and Ser^55^, were mutated to alanine and the effect on PknQ-mediated phosphorylation of MupFHA was subsequently analyzed. We found that the MupFHA^R41A^ and MupFHA^S55A^ mutants had an approximately 40% and 80% loss of phosphorylation signal compared to wild-type protein, respectively (Fig. 9D). These results indicate that FHA-mediated interaction of MupFHA is necessary for its phosphorylation by PknQ. This cooperation between FHA domain residues and other phosphorylated residues most likely helps in enhancing and regulating the interaction as well as phosphorylation of MupFHA. Importantly, these results are also in agreement with the docking studies where the Arg^41^ and Ser^55^ mutations weakened the interaction between MupFHA and PknQ (Fig. 5E and 5F).

### Autophosphorylation of PknQ is regulated by MupFHA

In the *M. tuberculosis* CDC1551 strain, the FHA domain containing protein EmbR2 (a structural homologue of EmbR) affects the kinase activity of PknH. Although EmbR2 is not phosphorylated by PknH, it inhibits the kinase autophosphorylation and inactivates the protein [55]. Similarly, Rv0020c, a FHA domain containing protein of *M. tuberculosis*, regulates the cell wall regulator pseudokinase MviN [17]. In our study, we found that MupFHA specifically inhibited PknQ activity in the *in vitro* kinase assay, indicating that their interaction may have a negative impact on the kinase activity (Fig. 8A). To further investigate the effect of the MupFHA interaction on PknQ activity, we used mass spectrometry to assess changes in phosphorylation patterns. PknQ was allowed to autophosphorylate in the presence of MupFHA and then analyzed by mass spectroscopy to determine the phosphorylation sites. Our results showed that PknQ only autophosphorylates on eight sites in the presence of MupFHA (Table 4) as compared to 20 sites in the absence of MupFHA (Table 1). These results confirm that MupFHA acts as a negative regulator of PknQ kinase activity. Interestingly, the phosphorylation of Thr^166^, Ser^170^ and Thr^174^ residues was impervious to MupFHA-based inhibition, underscoring the critical requirement of activation loop residues in the activity of PknQ. The kinase activation process involves initial phosphorylation on activation loop residues and subsequently other sites are phosphorylated to generate its active conformation. Inhibition by MupFHA may reduce/abrogate these conformational changes and therefore the kinase may only be able to reach its fully active conformation either in the absence of MupFHA or by any other ligand that might abolish this interaction.

**Table 4:** Phosphorylated residues of PknQ identified by *in vitro* kinase assays.

Phosphorylated tryptic peptide sequence of autophosphorylated PknQ	Phosphorylated residue(s)	Number of detected phosphate groups LC/MS/MS
EADLAA**pT**LSHPNIVTVFNR [60–78]	**T66**	1
AFDDT**pT**LTAIGSLVGTASYAAPEAIQGGSVDQR [159–191]	**T164**	1
AFDDT**pT**L**pT**AIGSLVGTASYAAPEAIQGGSVDQR [159–191]	**T164+T166**	2
AFDDT**pT**L**pT**AIG**S**LVG**T**ASYAAPEAIQGGSVDQR [159–191]	**T164+T166+(S170 or T174)**	3
IGSLVG**pT**ASYAAPEAIQGGSVDQR [168–191]	**T174**	1
FP**pT**AGALAGAAR [258–269]	**T260**	1
AALSGQPLPQAPPGGPK**pT**R [270–288]	**T287**	1
IWAAPPLSYP**pT**TRPPGI [289–305]	**T299**	1

Sequences of the phosphorylated peptides identified in autophosphorylated PknQ in the presence of MupFHA are indicated, as determined by mass spectrometry. Phosphorylated residues (pT or pS) are shown in bold.

Interestingly, Ser/Thr phosphatases are the only known negative regulators of STPK-mediated signaling and there is no such phosphatase encoding gene present on the pMUM001 plasmid. Therefore, MupFHA may help in regulating the activity of PknQ by limiting phosphorylation to specific substrate(s) and act as a principal controller of this signaling scheme. However, MupFHA may only act as an additional regulator of the kinase activity, while Ser/Thr phosphatase (Mul_0022) encoded in *M. ulcerans* genome may control the dephosphorylation as we found that MupFHA and PknQ get dephosphorylated by *M. tuberculosis* Ser/Thr phosphatase PstP (which is ∼94% similar to *M. ulcerans* PstP Mul_0022, Fig. S8).

### PknQ-mediated phosphorylation of MupDivIVA regulates its interaction with MupFHA

**Figure 10:**
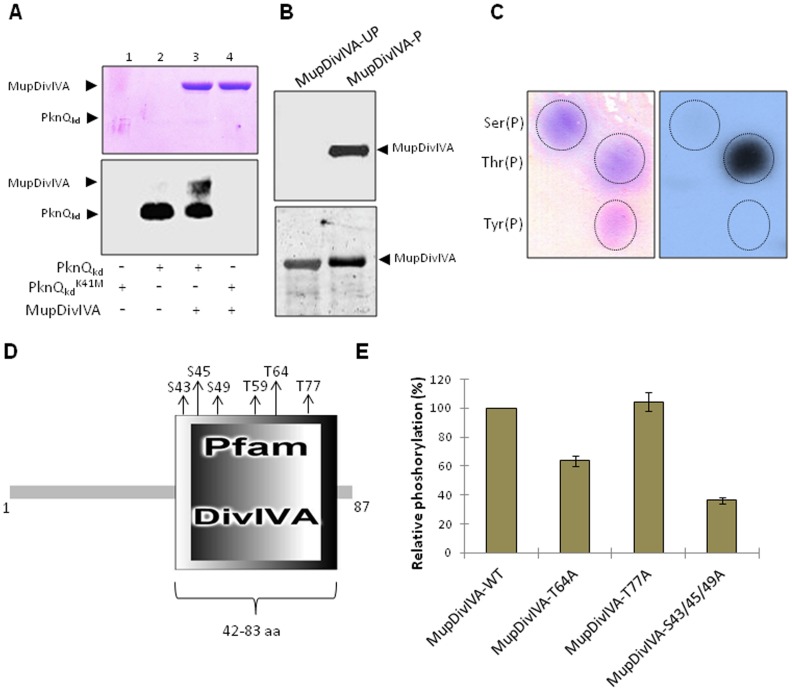
Phosphorylation of MupDivIVA by PknQ and interaction with MupFHA. (**A**) An *in vitro* kinase assay showing the phosphorylation of MupDivIVA by PknQ and PknQ^K41M^. MupDivIVA was phosphorylated by PknQ, while no phosphorylation was observed by PknQ^K41M^. The upper panel shows coomassie-stained SDS-PAGE and the lower panel shows the corresponding autoradiogram. (**B**) MupDivIVA was co-expressed with PknQ or PknQ^K41M^ in *E. coli* and the phosphorylation status of MupDivIVA was estimated using ProQ Diamond phosphoprotein staining. As shown in the upper panel, MupDivIVA co-expressed with PknQ was phosphorylated (MupDivIVA-P), while no phosphorylation was observed when it was co-expressed with PknQ^K41M^ (MupDivIVA-UP). The same gel was stained with Sypro Ruby stain (lower panel) to show equal loading of both samples. (**C**) PAA analysis of MupDivIVA phosphorylated by PknQ. The left panel shows ninhydrin-stained phosphoamino acid spots and the right panel shows the corresponding autoradiogram. Phosphorylation was detected on the spot corresponding to Thr(P), while minor phosphorylation was also seen on Ser(P). (**D**) Domain architecture of MupDivIVA analyzed by SMART domain prediction software. The phosphorylated residues (Ser^43^, Ser^45^, Ser^49^, Thr^59^, Thr^64^, and Thr^77^) are marked. (**E**) Relative phosphorylation of MupDivIVA phospho-site mutants using PknQ. Multiple mutants of MupDivIVA phosphorylation sites were generated and the loss in phosphorylation by PknQ was assessed. Phosphorylation on wild-type MupDivIVA was taken as 100% and relative phosphorylation was calculated. As shown in the histogram, maximum loss was observed in MupDivIVA^S43/45/49A^ triple mutant. The experiment was repeated three times and error bars show S.D. of three values.

STPKs regulate peptidoglycan synthesis and other cell wall processes in diverse bacteria [56]–[58]. In *M. tuberculosis*, PknA and PknB regulate Wag31, which is a DivIVA domain containing protein that regulates growth, morphology, polar cell wall synthesis, and peptidoglycan synthesis [59], [60]. In pMUM001, the kinase gene *mup011* (*pknQ*) and *mup012c* (encoding the DivIVA domain-containing protein MupDivIVA, Fig. S9) are adjacent. In earlier reports of *M. tuberculosis* STPKs, the proteins encoded by the neighboring genes of kinases were found to be specific substrates of those kinases [18], [39], [61]. To validate this hypothesis in the case of *M. ulcerans*, we determined if MupDivIVA was a PknQ substrate. The *mup012c* was cloned into pMAL-c2x and MupDivIVA was purified as a MBP-tagged fusion protein. We found that PknQ efficiently phosphorylated MupDivIVA in the *in vitro* kinase assay while there was no phosphorylation on MupDivIVA with PknQ^K41M^ (Fig. 10A). To test the authenticity of PknQ-dependent phosphorylation of MupDivIVA, we co-expressed PknQ or PknQ^K41M^ with MupDivIVA in the surrogate host *E. coli* using compatible expression vectors (pMAL-c2x-MupDivIVA and pACYCDuet1-PknQ). Phosphorylation-specific ProQ Diamond staining revealed that MupDivIVA was phosphorylated only when co-expressed with the catalytically active kinase (MupDivIVA-P) and not with the kinase-inactive mutant (MupDivIVA-UP) (Fig. 10B). PAA analysis showed that phosphorylation was located at serine and threonine residues of MupDivIVA (Fig. 10C). Mass spectrometry analysis of MupDivIVA identified six phosphorylation sites (Table 5). Incidentally, all phosphorylation sites were found to be localized within DivIVA core domain (42–83 aa) (Fig. 10D). Comparison of MupDivIVA phosphorylation sites with only phosphorylated threonine residue identified in *M. tuberculosis* Wag31 revealed that the three serine phosphorylation sites are present in the same DivIVA region in both the proteins (Fig. S9). Such strikingly similar phosphorylation patterns may indicate their conserved role. The three serine phosphorylation sites were mutagenized to alanine and were compared for their phosphorylation levels. The triple mutant MupDivIVA^S43/45/49A^ showed ∼70% loss of phosphorylation, indicating the importance of serine phosphorylation (Fig. 10E).

**Table 5:** Phosphorylated residues of MupDivIVA identified by *in vitro* kinase assay.

Phosphorylated tryptic peptide sequence of MupDivIVA phosphorylated by PknQ	Phosphorylated residue(s)	Number of detected phosphate groups LC/MS/MS
CAAPL**pT**R [54–60]	**T59**	1
CAAPL**pT**RGYDTESVDR [54–69]	**T59**	1
CAAPL**pT**RGYD**pT**ESVDR [54–69]	**T59+T64**	2
LIADELQGSDL**pS**ESDIHSITFR [32–53]	**S43**	1
LIADELQGSDLSE**pS**DIHSITFR [32–53]	**S45**	1
LIADELQGSDLSESDIH**pS**ITFR [32–53]	**S49**	1
GYD**pT**ESVDRFLDR [61–73]	**T64**	1
IAE**pT**IAR [74–80]	**T77**	1

Sequences of the phosphorylated peptides identified in MupDivIVA phosphorylated by PknQ are indicated, as determined by mass spectrometry. Phosphorylated residues (pT/pS) are shown in bold.

**Figure 11:**
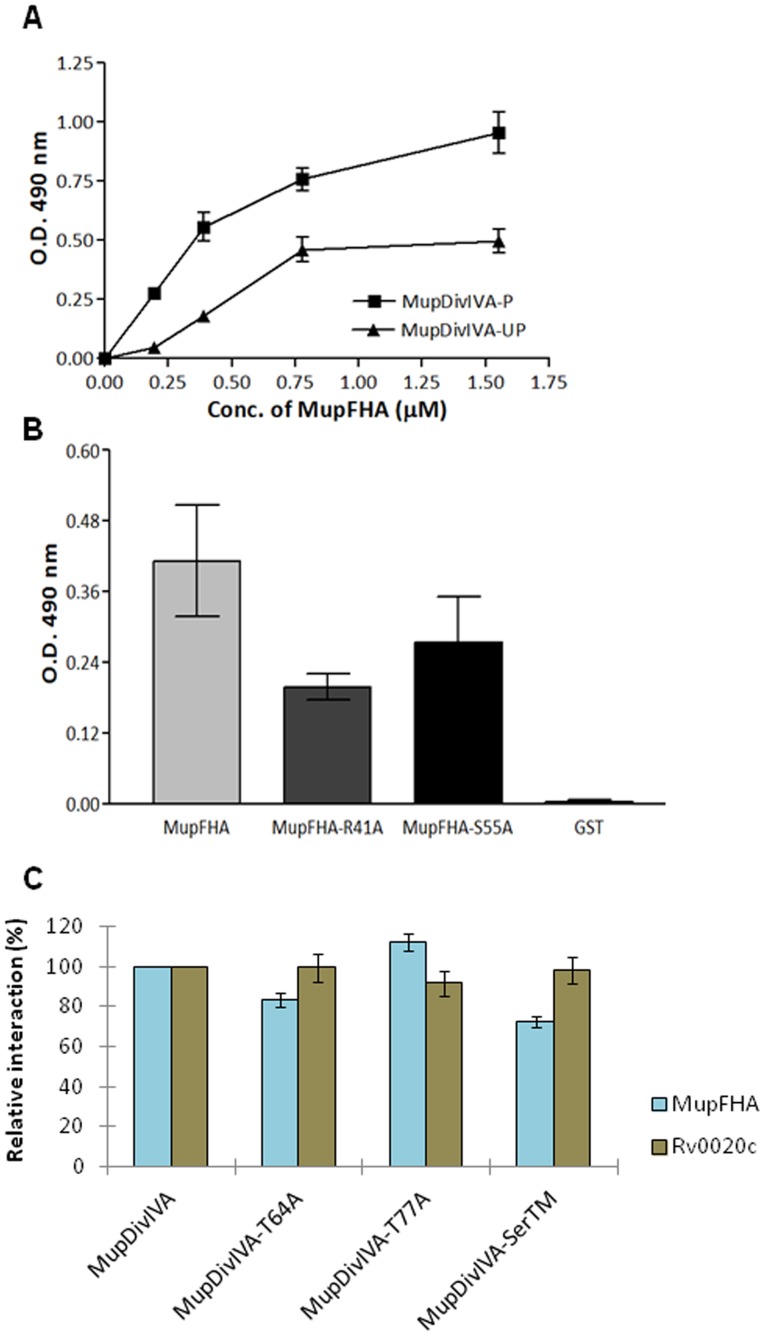
Interaction analysis of MupDivIVA and MupFHA. (**A**) *In vitro* interaction of MupFHA with MupDivIVA-P and MupDivIVA-UP by ELISA. As shown in the graph, MupFHA interacts profoundly with MupDivIVA-P, while weak interaction was observed with MupDivIVA-UP. The experiment was repeated three times and error bars show S.E. of three values. (**B**) Histogram showing ELISA-based comparative interaction of MupFHA and its mutants with MupDivIVA-P. The FHA domain mutants showed less interaction with MupDivIVA-P compared to the wild-type MupFHA. The experiment was repeated three times and error bars show S.E. of three values. (**C**) Relative interaction of MupFHA or Rv0020c with MupDivIVA and its mutants. ELISA was performed to study the interaction of MupDivIVA and its mutants with FHA domain containing proteins. Relative interaction was calculated considering the interaction of wild type MupDivIVA as 100% (for both MupFHA and Rv0020c). The triple mutant MupDivIVA^S43/45/49A^ (MupDivIVA-SerTM) shows decreased interaction with MupFHA but not with Rv0020c, thus signifying the binding of MupFHA (and not Rv0020c) with pSer residues. The experiment was repeated three times and error bars show S.E. of three values.

Since the FHA domain recognizes phosphorylated proteins, we hypothesized that MupFHA may interact with the phosphorylated MupDivIVA through a three-way regulatory process with PknQ. To evaluate this hypothesis, the MBP-tagged proteins MupDivIVA-P (phosphorylated) or MupDivIVA-UP (unphosphorylated) and GST-tagged MupFHA were used in a sandwich ELISA. We found that MupDivIVA phosphorylation increased its affinity for MupFHA (Fig. 11A). Furthermore, there was a considerable loss in interaction with MupDivIVA when the conserved FHA domain residues of MupFHA were mutated (Fig. 11B). These results confirm that the FHA domain mediates the interaction of MupFHA with phosphorylated MupDivIVA. Therefore, MupFHA interacts with MupDivIVA through a phosphorylation dependent manner, which is regulated by PknQ. Further, we used MupDivIVA to probe pSer binding affinity of MupFHA. We compared the interaction of MupDivIVA phosphorylation site mutants with MupFHA. *M. tuberculosis* Rv0020c was used to compare pThr specificity (Fig. 11C). This analysis revealed role of phosphorylated serine/threonine residues in MupFHA∶MupDivIVA interaction while only pThr residues regulate Rv0020c∶MupDivIVA interaction.

## Discussion

In this study we analyzed the STPK-mediated signaling system of *M. ulcerans*, which is the causative agent of Buruli ulcer. *M. ulcerans* is a slow growing bacterium (slower than *M. marinum* and *M. tuberculosis*) [62], [63] and this slow growth together with restrictive temperature requirements are the major reasons for our limited understanding about this important human pathogen and its signaling systems. Using *in silico* analysis, we identified 13 STPKs in the *M. ulcerans* genome that are distinct from its close relative *M. marinum* that has 24 STPKs [39]. STPKs of *M. tuberculosis* have been classified in five clades [39], and phylogenetic analysis reveals that *M. ulcerans* also has individual STPKs related to all five clades with an over-representation of the PknF/PknI/PknJ clade. Analyses of STPKs and FHA domain encoding genes confirmed that *M. ulcerans* underwent reductive evolution compared to *M. marinum*.

The presence of PknQ on the virulence-associated plasmid pMUM001 makes the *M. ulcerans* kinome exclusive than *M. tuberculosis* and other characterized bacterial kinomes [64]. Three such plasmids have been identified in the *Mycobacterium* species, including pMUM001 (*M. ulcerans* Agy 99), pMUM002 (*M. liflandii* 128FXT), and pMUM003 (*M. marinum* DL240490) [65]. These three plasmids are involved in mycolactone synthesis and most likely in pathogenesis. In addition, all of the plasmids encode a homolog of STPK (MUP011, MULP_022, and MUDP_075, respectively), although a frameshift mutation suggests that MUDP_075 is a pseudogene [65]. Notably, these plasmids have a conserved kinase locus (from the STPK gene [*mup011*] to the FHA domain-containing protein [*mup018c*]), although pMUM003 is slightly different, most likely due to a frameshift mutation [65]. Large plasmids, such as pMUM001, which encode proteins involved in adaptation to new environments, represent regular theme among many other bacterial pathogens, such as *B. anthracis*, *Y. pestis*, and *Shigella* [64], [66]–[68]. These species share a nearly identical genome structure and sequence with other species in their genera, but due to the acquisition of virulence-associated plasmids, these bacteria tend to acclimatize better to new conditions. In the plague causing bacteria *Y. pestis*, the virulence-associated plasmid pLB1 encodes a STPK YpkA that acts as a direct inducer of cell death by promoting apoptosis and actin depolymerization during infection [66], [69]. Interestingly, other than YpkA, pLB1 encodes several other antigens, and one of them, the type III secretion apparatus protein YscD also possesses an FHA domain [70]. The role of a virulence-associated plasmid in the pathogenesis of *M. ulcerans* has previously been described [4], [71], but its significance beyond mycolactone biosynthesis has not been appreciated until now. Our results demonstrated that multiple proteins encoded on pMUM001 are regulated by phosphorylation, and therefore this plasmid may be an important component of signaling cascades in *M. ulcerans*.

To understand the significance of PknQ in *M. ulcerans*, we elucidated the biochemical characteristics of this kinase. Our biochemical characterization identified both common and novel features. PknQ kinase activity is dependent on cofactors such as Fe^2+^, Mn^2+^, Mg^2+^, and Zn^2+^. Interestingly, the kinase is activated by Fe^2+^, while its activity is inhibited by Fe^3+^. This inhibition occurs in the presence of hemin, indicating that PknQ activity is regulated by iron and its redox state in the cellular milieu. The role of iron in the regulation of its kinase activity is justified by the presence of a FepB-like iron transporter domain at the C-terminus of PknQ. However, further studies are required to validate this aspect of PknQ signaling.

To understand the activation mechanism of PknQ, several mutants were generated. We found that the autophosphorylation of serine and threonine residues in the activation loop region regulates PknQ activity. In most *M. tuberculosis* STPKs, activity is regulated by two threonine residues present in the activation loop. Analogous residues (Thr^164^ and Thr^166^) are also phosphorylated in PknQ, but only Thr^166^ is critical for autophosphorylation activity. Furthermore, a novel serine residue, Ser^170^, was found to regulate the autokinase and substrate phosphorylation activities of PknQ. In fact, phosphorylation of activation loop residues is known to be required for stabilization of kinases as it can induce specific conformational changes important in substrate binding [72]. Thus, further structural studies on PknQ are needed to understand the role of each phosphoresidue and how this serine phosphorylation is mechanistically different from threonine phosphorylation.

We also identified the FHA-domain containing protein MupFHA as an interacting partner of PknQ. To understand how MupFHA and PknQ interact, we generated structural models to establish the basis of MupFHA and PknQ interaction. Interestingly, the PknQ and MupFHA interaction highlighted several novel aspects. The structural analyses suggest that PknQ-MupFHA interaction follows the canonical binding mode of pThr interaction with specific arginine and serine residues of FHA-domain. However, MupFHA additionally interacts with a pSer residue present in the activation loop of PknQ. The structural models were validated by experimental analysis using ELISA and affinity pull-down assays, thus establishing the structure-activity relationship for PknQ. Together, these findings highlighted the unconventional molecular recognition patterns of kinase∶FHA interactions.

The second unique aspect of the MupFHA∶PknQ interaction is the role of MupFHA as a negative regulator of PknQ. To the best of our knowledge, this is only the second report of any bacterial FHA-mediated regulation of the cognate kinase activity. It has been previously reported that EmbR2 inhibits PknH phosphorylation [55], but this is restricted to the *M. tuberculosis* CDC1551 strain. Nevertheless, MupFHA signaling is unique, as PknH does not phosphorylate EmbR2, while MupFHA is a substrate of PknQ. This regulation of kinase activity by FHA domains could have important implications in the spatio-temporal regulation of cellular signaling. It is important to note that the critical activation loop residues were phosphorylated even in the presence of MupFHA, thus indicating that MupFHA may only regulate the secondary phosphorylation sites and substrate binding of PknQ. To understand this inhibition further, we applied mass spectrometry and found a significant reduction in number of phosphorylation sites. However, the mass spectrometry analysis did not quantitate the phosphorylation stoichiometry of each site and we cannot rule out the possibility that the phosphorylation of key activation loop residues is also inhibited.

The third aspect of MupFHA signaling is the ability of both PknQ and MupFHA to interact with another phosphorylated protein, MupDivIVA. Our results indicate that PknQ phosphorylates MupDivIVA, which is a homolog of *M. tuberculosis* Wag31, another DivIVA domain-containing protein [60]. Wag31 in *M. tuberculosis* is already known to be phosphorylated by PknB and PknA and this phosphorylation is critical for cell growth and peptidoglycan synthesis [59]. Upon phosphorylation, MupDivIVA also interacts with MupFHA in a phosphorylation-dependent manner. Therefore, this study provides insights into three-way regulation involving dynamic signaling between PknQ, MupFHA, and MupDivIVA.

In conclusion, our study is the first analysis of signaling pathways in *M. ulcerans* and has revealed many novel aspects of signaling systems among *Mycobacterium* species. Our results indicate that PknQ could be an important sensor of extracellular cues in *M. ulcerans* and can propagate the signals to MupFHA and MupDivIVA. Moreover, iron and MupFHA may act as quenchers in this phosphorylation cascade. Taken together, our data underscore the importance of structure-activity studies in unraveling the PknQ-MupFHA signaling axis in *M. ulcerans* and provide an interesting starting point to work towards understanding this pathogen.

## Supporting Information

Figure S1 **Domain analysis of** ***M. ulcerans*** **STPKs.** Since the STPKs are not characterized, we used SMART domain analysis to predict the possible domains present in these STPKs on the basis of their conserved protein sequences. PknQ possesses N-terminal catalytic domain and C-terminal extracellular region containing a periplasmic-binding domain, which also contains sequences conserved for FepB (such as iron transporter domain).
(TIF)

Figure S2 **PknQ domain organization.** Multiple sequence alignment showing conserved Hank's subdomains present in PknQ. The alignment was done with *M. tuberculosis* STPKs PknB and PknJ. The conserved subdomain sequences have been highlighted (yellow) and the corresponding domains have been marked.
(TIF)

Figure S3 **Relative phosphorylation efficiencies of PknQ and its mutants.** (**A**) Histogram shows relative phosphorylation considering the intensity of PknQ^WT^ as 100%. Corresponding autoradiogram (top) and SDS-PAGE image (lower) is shown. (**B**) Histogram shows relative phosphorylation of PknQ phosphorylation site mutants, considering the intensity of PknQ as 100%. The experiment was repeated three times and error bars represent S.D. of three values.
(TIF)

Figure S4 **Multiple sequence alignment of MupFHA.** Multiple sequence alignment of amino acid sequences of *M. ulcerans* MupFHA, *M. tuberculosis* Rv0020c and Human enzymes Rad53-FHA1, Rad53-FHA2 and polynucleotide kinase (PNK). MupFHA Residues corresponding to the five most conserved FHA-domain residues are colored red. Additional MupFHA residues that are involved in binding with PknQ are colored green.
(TIF)

Figure S5 **Docking analysis of MupFHA with PknQ activation loop.** The structure of PknQ phosphorylated at Ser^170^ and Thr^174^ was docked with MupFHA. As clearly evident, Thr^166^ does not interact with MupFHA. Figure A shows the whole complex and B shows enlarged section of interaction locus.
(TIF)

Figure S6 **Interaction of MupFHA and PknQ in the presence of phospho-peptides.** Treatment of MupFHA with pThr or pSer peptides saturates the phosphoprotein binding sites of the FHA domain. Therefore, PknQ and MupFHA show decreased interaction after the phospho-peptide treatment as most of the phosphoprotein binding sites are already saturated. Relative interaction values were calculated considering 100% interaction in absence of any peptide. Addition of pSer or pThr peptides leads to decrease in interaction and thus proves phospho-specific affinity of MupFHA.
(TIF)

Figure S7 **Relative interaction of MupFHA with PrkD and its mutant PrkD^S162A^.** ELISA was used to demonstrate the interaction of these proteins and relative interaction was calculated considering the interaction of PrkD^wt^ as 100%. PrkD^S162A^ shows considerably less interaction with MupFHA.
(TIF)

Figure S8 **Role of Ser/Thr phosphatase.** (**A**) Multiple sequence alignment (clustalW) of *M. ulcerans* Ser/Thr phosphatase (Mul_pstP) with *M. tuberculosis* phosphatase (Mtb_pstP). The alignment shows >90% sequence identity. (**B**) Dephosphorylation of PknQ and MupFHA by *M. tuberculosis* PstP. Autoradiogram (lower panel) shows loss in phosphorylation signal in presence of PstP. Corresponding SDS-PAGE image is shown in upper panel.
(TIF)

Figure S9 **Multiple sequence alignment (clustalW) of** ***M. ulcerans*** **MupDivIVA with** ***M. tuberculosis*** **Wag31.** The phosphorylated residues are highlighted in both the sequences. As shown in the alignment, the 3 phosphorylated serine residues of MupDivIVA are localized in the same region as that of the only phosphorylation site of Wag31.
(TIF)

Table S1 **Primers and clones used in the study.**
(DOCX)

Movie S1 **Movie of the PknQ-pSer170/pThr174 and FHA domain complex simulation for 10 ns (stick representation).**
(MP4)

Movie S2 **Movie of the PknQ-pSer170/Thr174Ala and FHA domain complex simulation for 10 ns (stick representation).**
(MP4)

Movie S3 **Movie of the PknQ-Ser170Ala/pThr174 and FHA domain complex simulation for 10 ns (stick representation).**
(MP4)
