# Supplementary figures and images for: Identification of Ser/Thr kinase and Forkhead Associated Domains in Mycobacterium ulcerans: Characterization of Novel Association between Protein Kinase Q and MupFHA
Source: PLoS Negl Trop Dis. 2014 Nov 20;8(11):e3315. doi: 10.1371/journal.pntd.0003315 (PMC4238996; doi:10.1371/journal.pntd.0003315)

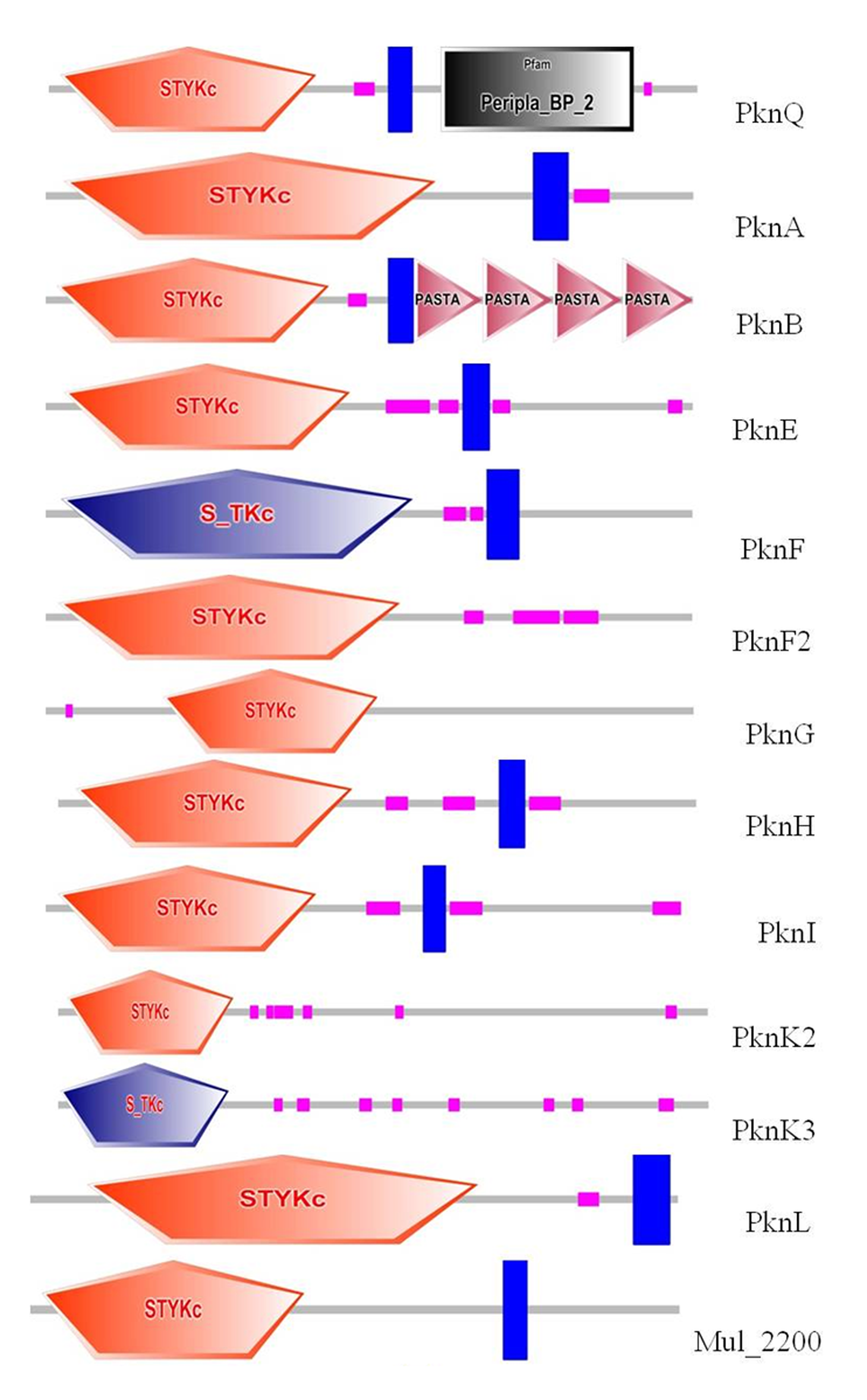

Supplement: Figure S1 — Domain analysis of M. ulcerans STPKs. Since the STPKs are not characterized, we used SMART domain analysis to predict the possible domains present in these STPKs on the basis of their conserved protein sequences. PknQ possesses N-terminal catalytic domain and C-terminal extracellular region containing a periplasmic-binding domain, which also contains sequences conserved for FepB (such as iron transporter domain). (TIF) [file pntd.0003315.s001.tif]

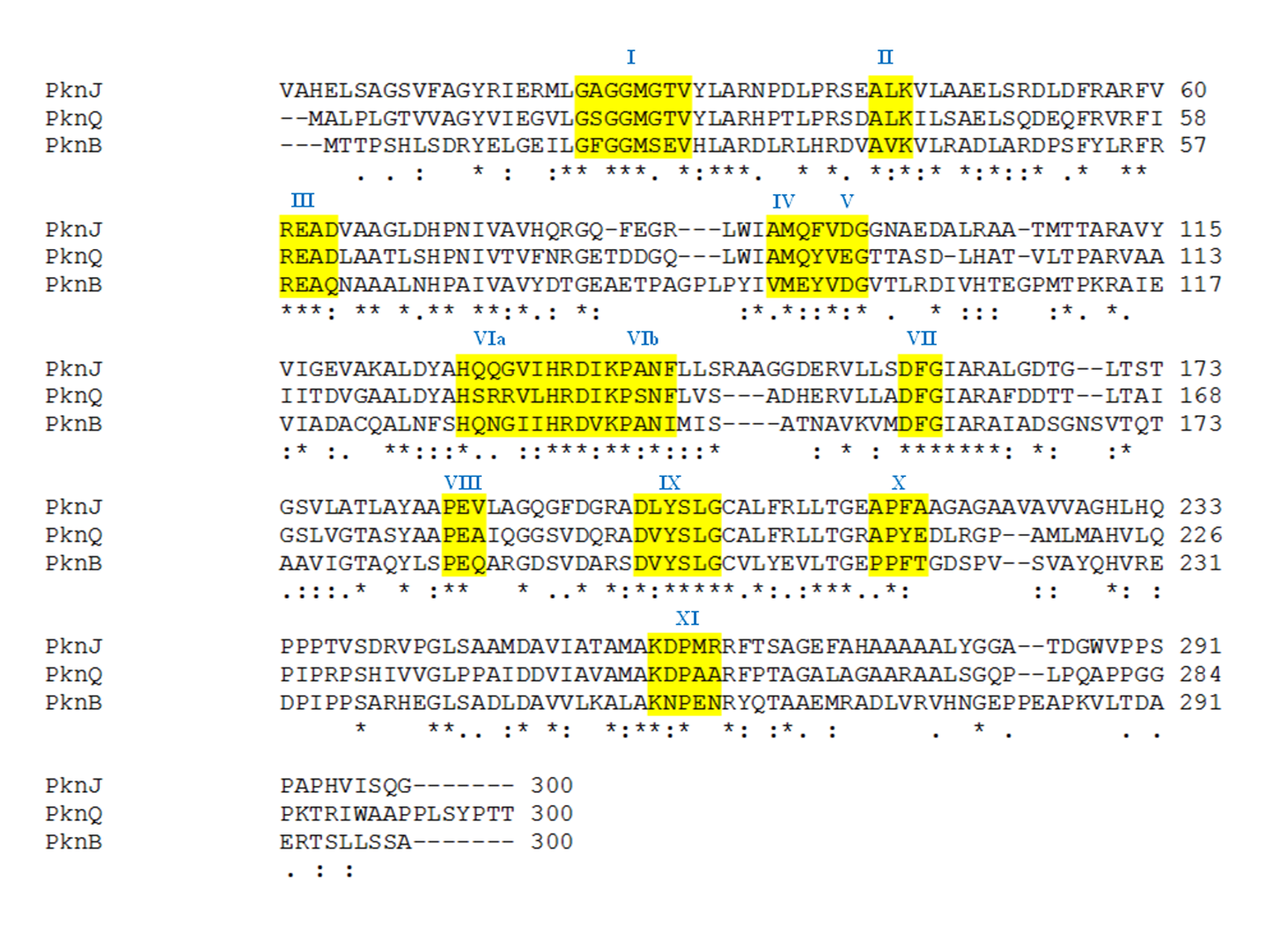

Supplement: Figure S2 — PknQ domain organization. Multiple sequence alignment showing conserved Hank's subdomains present in PknQ. The alignment was done with M. tuberculosis STPKs PknB and PknJ. The conserved subdomain sequences have been highlighted (yellow) and the corresponding domains have been marked. (TIF) [file pntd.0003315.s002.tif]

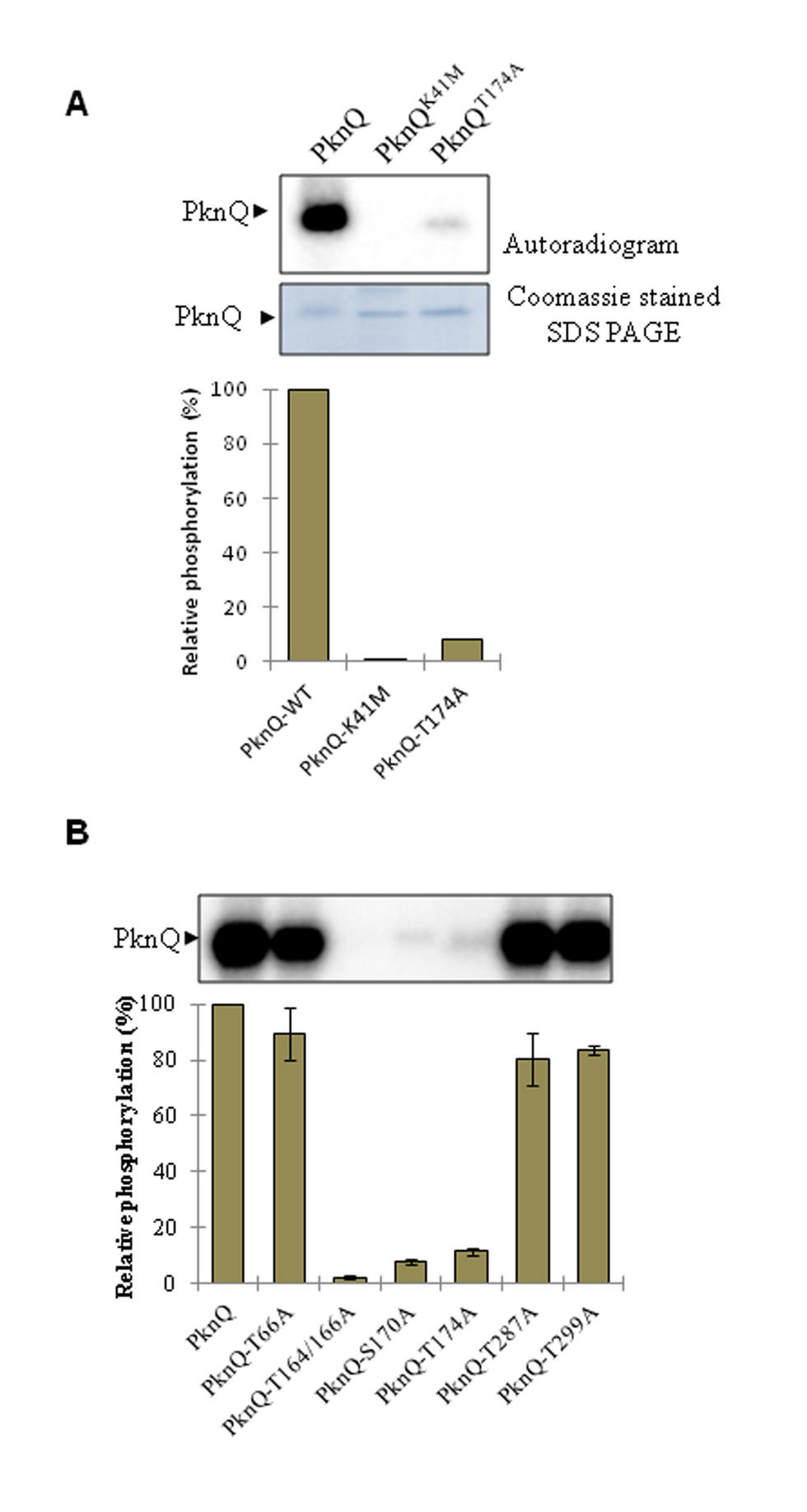

Supplement: Figure S3 — Relative phosphorylation efficiencies of PknQ and its mutants. (A) Histogram shows relative phosphorylation considering the intensity of PknQWT as 100%. Corresponding autoradiogram (top) and SDS-PAGE image (lower) is shown. (B) Histogram shows relative phosphorylation of PknQ phosphorylation site mutants, considering the intensity of PknQ as 100%. The experiment was repeated three times and error bars represent S.D. of three values. (TIF) [file pntd.0003315.s003.tif]

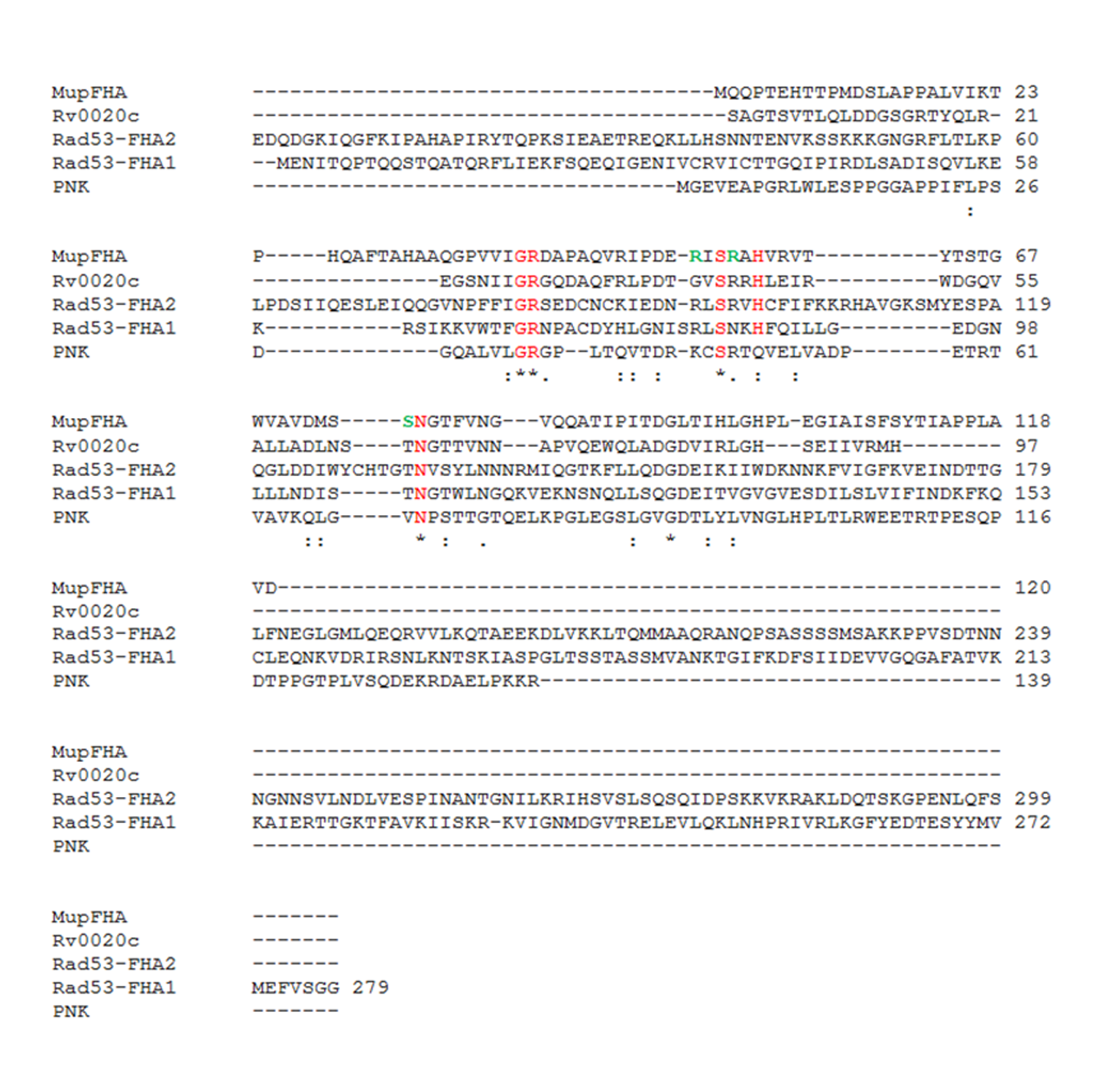

Supplement: Figure S4 — Multiple sequence alignment of MupFHA. Multiple sequence alignment of amino acid sequences of M. ulcerans MupFHA, M. tuberculosis Rv0020c and Human enzymes Rad53-FHA1, Rad53-FHA2 and polynucleotide kinase (PNK). MupFHA Residues corresponding to the five most conserved FHA-domain residues are colored red. Additional MupFHA residues that are involved in binding with PknQ are colored green. (TIF) [file pntd.0003315.s004.tif]

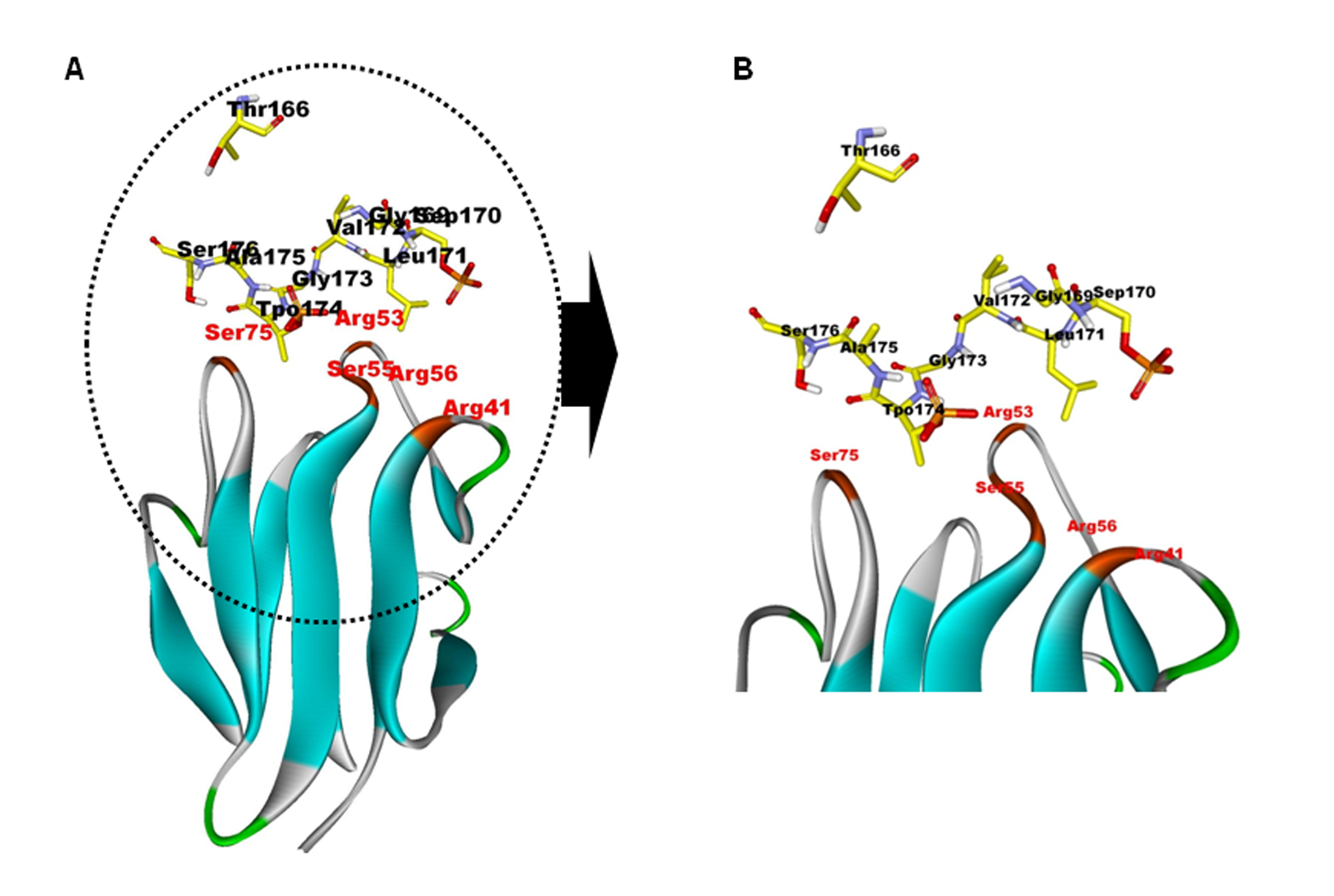

Supplement: Figure S5 — Docking analysis of MupFHA with PknQ activation loop. The structure of PknQ phosphorylated at Ser170 and Thr174 was docked with MupFHA. As clearly evident, Thr166 does not interact with MupFHA. Figure A shows the whole complex and B shows enlarged section of interaction locus. (TIF) [file pntd.0003315.s005.tif]

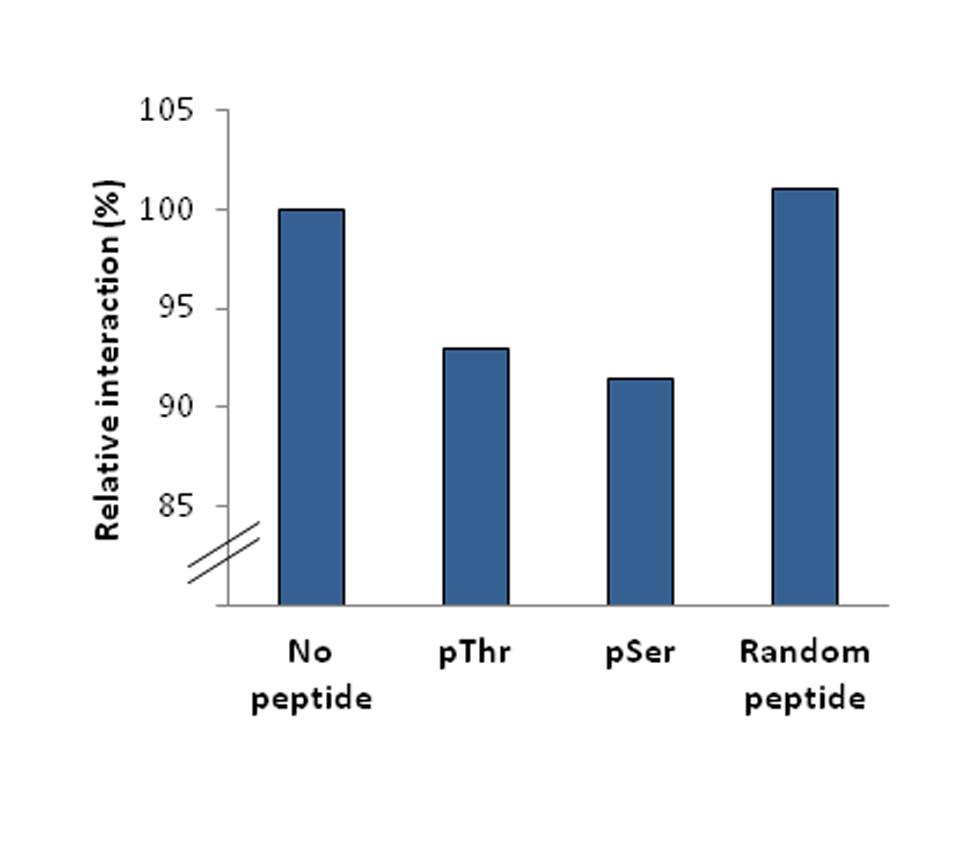

Supplement: Figure S6 — Interaction of MupFHA and PknQ in the presence of phospho-peptides. Treatment of MupFHA with pThr or pSer peptides saturates the phosphoprotein binding sites of the FHA domain. Therefore, PknQ and MupFHA show decreased interaction after the phospho-peptide treatment as most of the phosphoprotein binding sites are already saturated. Relative interaction values were calculated considering 100% interaction in absence of any peptide. Addition of pSer or pThr peptides leads to decrease in interaction and thus proves phospho-specific affinity of MupFHA. (TIF) [file pntd.0003315.s006.tif]

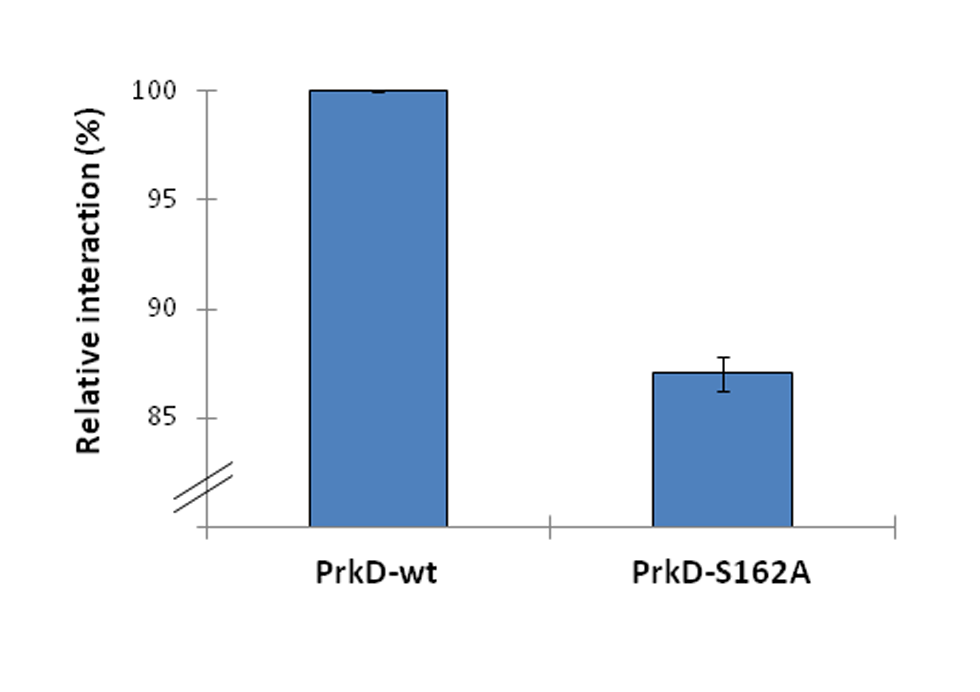

Supplement: Figure S7 — Relative interaction of MupFHA with PrkD and its mutant PrkDS162A. ELISA was used to demonstrate the interaction of these proteins and relative interaction was calculated considering the interaction of PrkDwt as 100%. PrkDS162A shows considerably less interaction with MupFHA. (TIF) [file pntd.0003315.s007.tif]

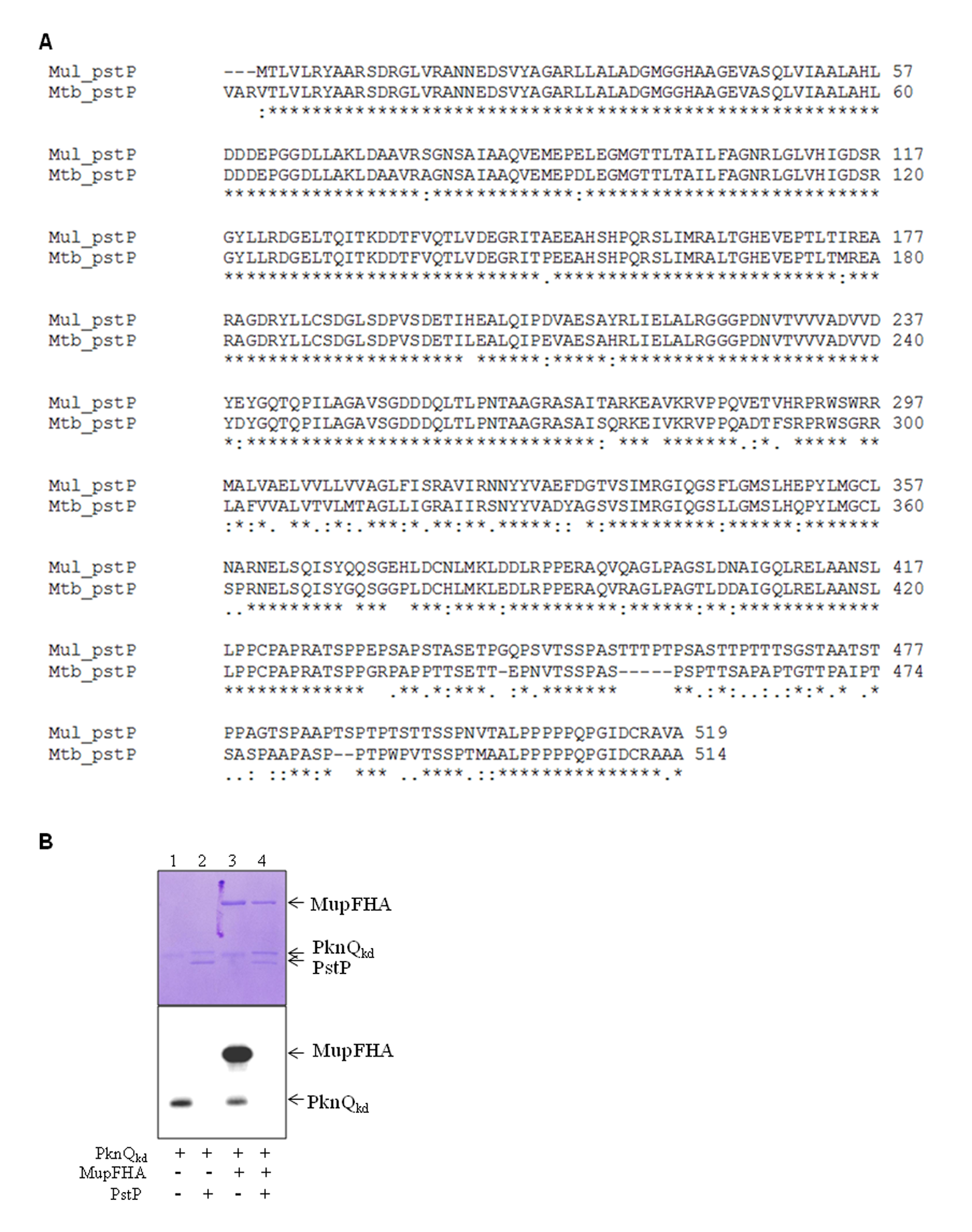

Supplement: Figure S8 — Role of Ser/Thr phosphatase. (A) Multiple sequence alignment (clustalW) of M. ulcerans Ser/Thr phosphatase (Mul_pstP) with M. tuberculosis phosphatase (Mtb_pstP). The alignment shows >90% sequence identity. (B) Dephosphorylation of PknQ and MupFHA by M. tuberculosis PstP. Autoradiogram (lower panel) shows loss in phosphorylation signal in presence of PstP. Corresponding SDS-PAGE image is shown in upper panel. (TIF) [file pntd.0003315.s008.tif]

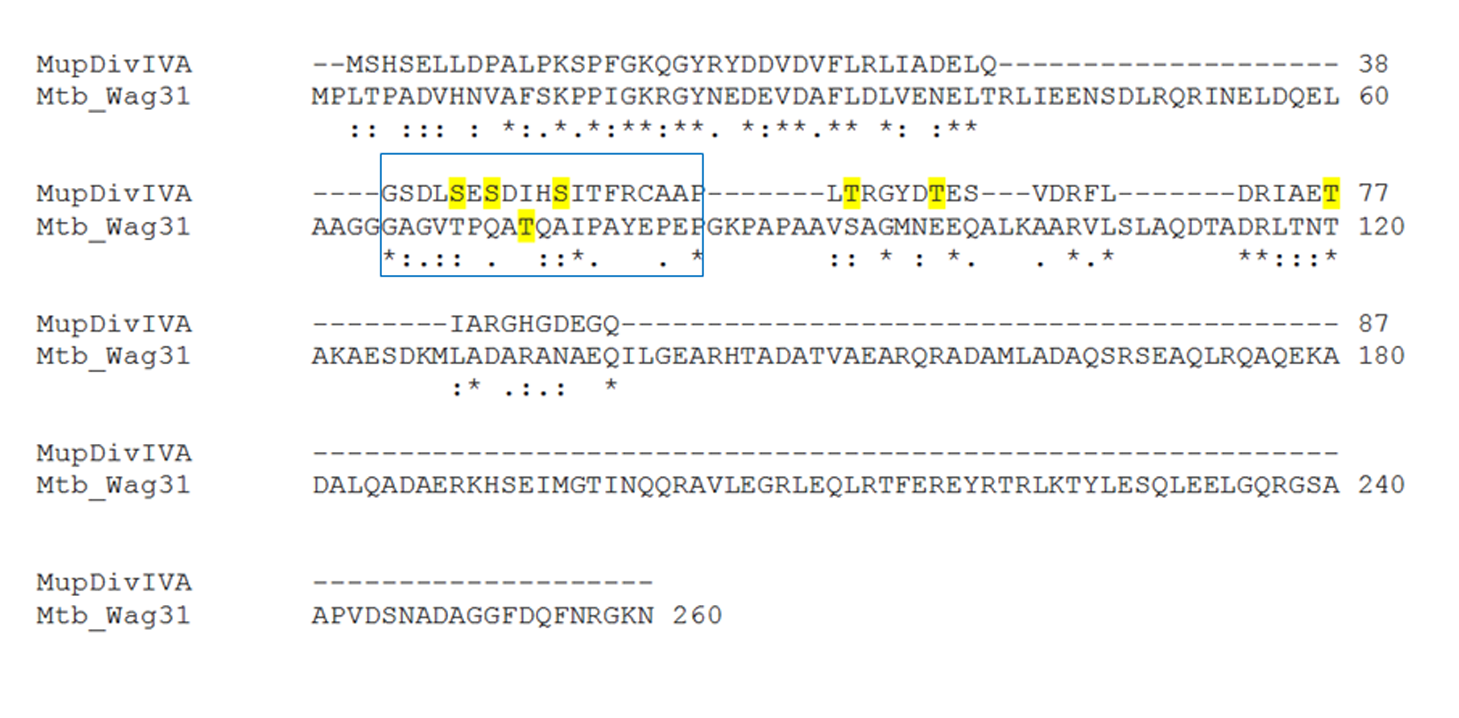

Supplement: Figure S9 — Multiple sequence alignment (clustalW) of M. ulcerans MupDivIVA with M. tuberculosis Wag31. The phosphorylated residues are highlighted in both the sequences. As shown in the alignment, the 3 phosphorylated serine residues of MupDivIVA are localized in the same region as that of the only phosphorylation site of Wag31. (TIF) [file pntd.0003315.s009.tif]
