# Supplementary material for: Identification of Ser/Thr kinase and Forkhead Associated Domains in Mycobacterium ulcerans: Characterization of Novel Association between Protein Kinase Q and MupFHA
Source: PLoS Negl Trop Dis. 2014 Nov 20;8(11):e3315. doi: 10.1371/journal.pntd.0003315 (PMC4238996; doi:10.1371/journal.pntd.0003315)
Supplement: Table S1 — Primers and clones used in the study. (DOCX) [file pntd.0003315.s010.docx]

**Table S1: Primers and clones used in the study.**

| Genes  (protein size*) | Vector | Restriction sites | Primers (5′🡪3′)** |
| --- | --- | --- | --- |
| *pknQ_fl_*  (1-660 aa, 73 kDa) | pPro-Ex-HTc | FP-EcoRI | GCCATGGGGATCCGGAATTCCGTGGCGTTGCCTTTAGGAAC |
|  |  | RP-XhoI | GCTTGGTACCGCATGCCTCGAGTCAGTGGATCTGGTGGGCGAGTT |
|  | pACYCDuet-1 | FP-NdeI | GACGGGGAAGTACATATGGCCGTGGCGTTGCC |
|  |  | RP-XhoI | GCCAGTAACGGCGATACACTCGAGTCAGTGGATC |
| *pknQ_kd_*  (1-340 aa, 38 kDa) | pPro-Ex-HTc | FP-EcoRI | GCCATGGGGATCCGGAATTCCGTGGCGTTGCCTTTAGG |
|  |  | RP-NotI | CCCCATTGCGGCCGCGAAGCTTTCGCTTGCGCCGGCG |
| *pknQ^K41M^* (1-340 aa, 38 kDa) | pPro-Ex-HTc, pACYCDuet-1 | FP | CCGCGCTCTGATGCCCTCATGATCTTGTCCGCCGAGCTG |
|  |  | RP | CAGCTCGGCGGACAAGATCATGAGGGCATCAGAGCGCGG |
| *mup018c* (1-362 aa, 40 kDa) | pGEX-5X-3 | FP-BamHI | CAGGGCGCCATGGGGATCCACATGCAACAGCCCACAGAGCACACG |
|  |  | RP-XhoI | GCTTGGTACCGCATGCCTCGAGTCAGCGCTGACTCAGCAGTGATGTC |
| *mup012c* (1-87 aa, 9.6 kDa) | pMAL-c2x | FP-BamHI | GGACCTTGAGTTCGGATCCGTGAGCCACAGTG |
|  |  | RP-BamHI | CACTGACCCGGCTGTGGATCCGTTACTGGCCCTCGTC |
| *mup012c^T64A^* (1-87 aa, 9.6 kDa) | pMAL-c2x | FP | GACGCGCGGATACGACGCTGAGTCAGTCGATCG |
|  |  | RP | CGATCGACTGACTCAGCGTCGTATCCGCGCGTC |
| *mup012c^T77A^* (1-87 aa, 9.6 kDa) | pMAL-c2x | FP | CTTGGATCGCATCGCAGAAGCGATCGCTCGAGGTCATGG |
|  |  | RP | CCATGACCTCGAGCGATCGCTTCTGCGATGCGATCCAAG |
| *mup012c^S43/45/49A^* (1-87 aa, 9.6 kDa) | pMAL-c2x | FP | GGCAGCGACCTTGCTGAGGCCGACATCCACGCCATCACGTTCCG |
|  |  | RP | CGGAACGTGATGGCGTGGATGTCGGCCTCAGCAAGGTCGCTGCC |
| *mup018c^R41A^* (1-362 aa, 40 kDa) | pGEX-5X-3 | FP | GGCCGGTCGTGATCGGCGCCGACGCTCCGGCCCAGG |
|  |  | RP | CCTGGGCCGGAGCGTCGGCGCCGATCACGACCGGCC |
| *mup018c^S55A^* (1-362 aa, 40 kDa) | pGEX-5X-3 | FP | CATCCCCGACGAGCGGATTGCGCGCGCCCACGTTCGGG |
|  |  | RP | CCCGAACGTGGGCGCGCGCAATCCGCTCGTCGGGGATG |
| *mup018c^T8A^* (1-362 aa, 40 kDa) | pGEX-5X-3 | FP | CAACAGCCCACAGAGCACGCGACGCCAATGGATTCTTTG |
|  |  | RP | CAAAGAATCCATTGGCGTCGCGTGCTCTGTGGGCTGTTG |
| *mup018c^T210A^* (1-362 aa, 40 kDa) | pGEX-5X-3 | FP | CAGCCCGACGAAGAGCGCGCCCACGTCTTGACCGCCAC |
|  |  | RP | GTGGCGGTCAAGACGTGGGCGCGCTCTTCGTCGGGCTG |
| *mup018c^T123A^* (1-362 aa, 40 kDa) | pGEX-5X-3 | FP | CTGGCTGTCGACGATGCCGCCGAACACATCGAAGAC |
|  |  | RP | GTCTTCGATGTGTTCGGCGGCATCGTCGACAGCCAG |
| *mup018c^T214A^* (1-362 aa, 40 kDa) | pGEX-5X-3 | FP | GAGCGCACCCACGTCTTGGCCGCCACCACTCCGGTGC |
|  |  | RP | GCACCGGAGTGGTGGCGGCCAAGACGTGGGTGCGCTC |
| *pknQ^T164A^* (1-340 aa, 38 kDa) | pPro-Ex-HTc | FP | CCGCGCCTTTGACGACACCGCGTTGACCGCAATCGGCTC |
|  |  | RP | GAGCCGATTGCGGTCAACGCGGTGTCGTCAAAGGCGCGG |
| *pknQ^T166A^* (1-340 aa, 38 kDa) | pPro-Ex-HTc | FP | CTTTGACGACACCACGTTGGCCGCAATCGGCTCGTTGGTC |
|  |  | RP | GACCAACGAGCCGATTGCGGCCAACGTGGTGTCGTCAAAG |
| *pknQ^T164/166A^* (1-340 aa, 38 kDa) | pPro-Ex-HTc | FP | GCGCCTTTGACGACACCGCGTTGGCCGCAATCGGCTCGTTG |
|  |  | RP | CAACGAGCCGATTGCGGCCAACGCGGTGTCGTCAAAGGCGC |
| *pknQ^S170A^* (1-340 aa, 38 kDa) | pPro-Ex-HTc | FP | CGTTGACCGCAATCGGCGCGTTGGTCGGCACCGCC |
|  |  | RP | GGCGGTGCCGACCAACGCGCCGATTGCGGTCAACG |
| *pknQ^T174A^* (1-340 aa, 38 kDa) | pPro-Ex-HTc | FP | CGGCTCGTTGGTCGGCGCCGCCTCCTACGCCGC |
|  |  | RP | GCGGCGTAGGAGGCGGCGCCGACCAACGAGCCG |
| *pknQ^T260A^* (1-340 aa, 38 kDa) | pPro-Ex-HTc | FP | CCGCCGCCCGATTCCCCGCGGCCGGGGCACTCGCC |
|  |  | RP | GGCGAGTGCCCCGGCCGCGGGGAATCGGGCGGCGG |
| *pknQ^T66A^* (1-340 aa, 38 kDa) | pPro-Ex-HTc | FP | GCGAAGCCGACCTGGCCGCGGCACTGAGCCACCCCAATATC |
|  |  | RP | GATATTGGGGTGGCTCAGTGCCGCGGCCAGGTCGGCTTCGC |
| *pknQ^T287A^* (1-340 aa, 38 kDa) | pPro-Ex-HTc | FP | CACCCGGCGGGCCGAAAGCCAGGATTTGGGCGGCCCC |
|  |  | RP | GGGGCCGCCCAAATCCTGGCTTTCGGCCCGCCGGGTG |
| *pknQ^T299A^* (1-340 aa, 38 kDa) | pPro-Ex-HTc | FP | CGCCGCTGTCATATCCGGCCACGCGACCCCCCGGG |
|  |  | RP | CCCGGGGGGTCGCGTGGCCGGATATGACAGCGGCG |
| Rv0020c (1-527 aa, 57 kDa) | pPro-Ex-HTc | FP-EcoRI | GTGAGGCGAGCGCTGAATTCAATGGGTAGCCAGAAAAGGCT |
|  |  | RP-NotI | CCCCGGCAGCTTCGAGCGGCCGCTTCAGTGCATGCGGACG |

* Protein size is shown without recombinant tag

** Restriction sites/mutated residues have been underlined.
